# Density Functional Prediction of Quasiparticle, Excitation, and Resonance Energies of Molecules With a Global Scaling Correction Approach

**DOI:** 10.3389/fchem.2020.588808

**Published:** 2020-12-08

**Authors:** Xiaolong Yang, Xiao Zheng, Weitao Yang

**Affiliations:** ^1^Hefei National Laboratory for Physical Sciences at the Microscale and Synergetic Innovation Center of Quantum Information and Quantum Physics, University of Science and Technology of China, Hefei, China; ^2^Department of Chemistry, Duke University, Durham, NC, United States; ^3^Key Laboratory of Theoretical Chemistry of Environment, School of Chemistry and Environment, South China Normal University, Guangzhou, China

**Keywords:** density functional theory, delocalization error, scaling correction approach, quasiparticle energies, electronic excitation energies, electron affinity

## Abstract

Molecular quasiparticle and excitation energies determine essentially the spectral characteristics measured in various spectroscopic experiments. Accurate prediction of these energies has been rather challenging for ground-state density functional methods, because the commonly adopted density function approximations suffer from delocalization error. In this work, by presuming a quantitative correspondence between the quasiparticle energies and the generalized Kohn–Sham orbital energies, and employing a previously developed global scaling correction approach, we achieve substantially improved prediction of molecular quasiparticle and excitation energies. In addition, we also extend our previous study on temporary anions in resonant states, which are associated with negative molecular electron affinities. The proposed approach does not require any explicit self-consistent field calculation on the excited-state species, and is thus highly efficient and convenient for practical purposes.

## 1. Introduction

Density function theory (DFT) (Hohenberg and Kohn, 1964) has made great success in practical calculations for ground-state electronic properties because of its outstanding balance between accuracy and computational cost. In the Kohn–Sham (KS) scheme of DFT (Hohenberg and Kohn, 1964; Kohn and Sham, 1965), the effective single-particle equations can be written as (by omitting the spin indices and adopting the atomic units)

(1) $$\begin{array}{l} \left[-\frac{1}{2}\nabla^{2}+v_{\text{H}}\left(\text{r}\right)+v_{\text{ext}}\left(\text{r}\right)+v_{\text{xc}}\left(\text{r}\right)\right]\phi_{m}\left(\text{r}\right)=\varepsilon_{m}\phi_{m}\left(\text{r}\right). \end{array}$$

Here, *v*_ext_(**r**) is the external potential, *v*_H_(**r**) is the Hartree potential, *v*_xc_(**r**) is the local exchange-correlation (XC) potential, and {ϕ_*m*_(**r**)} and {ε_*m*_} are the KS/generalized KS (GKS) orbitals and their eigenvalues, respectively. In the GKS scheme, *v*_xc_(**r**) is replaced by a non-local potential, $v_{\text{xc}}\left(\text{r},{\text{r}}^{\prime }\right)$. The KS equations can be solved self-consistently to produce the ground-state energy and charge density. However, it is challenging to apply conventional ground-state density functional methods to calculate excited-state-related properties, such as the quasiparticle (QP) energies and the electronic excitation energies, which will be introduced as follows.

When an electronic system is perturbed by incoming photons or electrons, in order to preserve a single-particle picture, the concept of QP is often adopted. In a direct photoemission experiment, an electron on a molecule absorbs the energy of a photon and gets excited away from the molecule. Such a process leaves a quasihole in the molecule whose energy level is renormalized by the presence of the other electrons. Similarly, in an inverse photoemission experiment, an electron attaches to a molecule by emitting a photon, which leads to the formation of a quasielectron whose energy level is influenced by the existing electrons in the molecule (Onida et al., 2002).

The actual QP energies and wavefunctions can be obtained by solving the QP equations as follows (Hedin, 1965; Aulbur et al., 2000),

(2) $$\begin{array}{r} \left[-\frac{1}{2}\nabla^{2}+v_{\text{H}}\left(\text{r}\right)+v_{\text{ext}}\left(\text{r}\right)\right]\psi_{m}\left(\text{r}\right)\\ +\int \Sigma \left(\text{r},{\text{r}}^{\prime };\omega_{m}\right)\psi_{m}\left({\text{r}}^{\prime }\right)d{\text{r}}^{\prime }=\omega_{m}\psi_{m}\left(\text{r}\right). \end{array}$$

Here, {ψ_*m*_(**r**)} and {ω_*m*_} are the QP wavefunctions and energies, respectively, and Σ is a non-local and energy-dependent self-energy operator, with the imaginary part of its eigenvalues giving the lifetime of the QPs. To enable practical calculations, an approximate scheme for Σ is to be employed. The most widely used scheme is the *GW* approximation (Hedin, 1965; Hybertsen and Louie, 1986; Aulbur et al., 2000; Dvorak et al., 2014). Therefore, regarding the calculation of QP energies, many-body perturbation theory within the *GW* approximation has become a popular method at present (Hedin, 1965; Hybertsen and Louie, 1986; Louie and Hybertsen, 1987; Aulbur et al., 2000; Onida et al., 2002; Dvorak et al., 2014). However, the somewhat large computational cost makes it difficult to apply the *GW* method to complex systems. Thus, a highly efficient and accurate method for the prediction of QP energies is sought for.

It is tempting to relate the KS/GKS orbital energies to QP energies, because the KS and GKS schemes are in conformity with an effective single-electron description. However, with conventional density functional approximations (DFAs), such as the local density approximation (LDA) (Slater, 1951; Vosko et al., 1980), generalized gradient approximations (GGAs), and hybrid functionals, the calculated KS/GKS orbital energies usually deviate severely from the QP energies. Such deviations have also led to significant underestimation of band gaps, which is largely due to the delocalization error associated with the DFAs (Cohen et al., 2008a). In the exact DFT, the ground-state energy of a system with a fractional number of electrons, *E*_0_(*N*+*n*) (subscript 0 denotes the ground state corresponding to the fixed *v*_ext_), should satisfy the Perdew–Parr–Levy–Balduz (PPLB) condition (Perdew et al., 1982, 2007; Yang et al., 2000): *E*_0_(*N* + *n*) = (1 − *n*)*E*_0_(*N*) + *nE*_0_(*N* + 1), where 0 < *n* < 1 is a fractional number.

The PPLB condition infers that ${\left(\frac{\partial E_{0}}{\partial N}\right)}_{-}=-I$ and ${\left(\frac{\partial E_{0}}{\partial N}\right)}_{+}=-A$, where *I* ≡ *E*_0_(*N* − 1) − *E*_0_(*N*) and *A* ≡ *E*_0_(*N*) − *E*_0_(*N* + 1) are the vertical ionization potential (IP) and electron affinity (EA) of the *N*-electron system, respectively. It has been proved (Cohen et al., 2008a; Yang et al., 2012) that if the XC energy is an explicit and differentiable functional of the electron density or the KS reduced density matrix, we have ${\left(\frac{\partial E_{0}}{\partial N}\right)}_{-}=\varepsilon_{\text{HOMO}}$ and ${\left(\frac{\partial E_{0}}{\partial N}\right)}_{+}=\varepsilon_{\text{LUMO}}$, where ε_HOMO_ and ε_LUMO_ are the energies of the highest occupied molecular orbital (HOMO) and the lowest unoccupied molecular orbital (LUMO), respectively. Therefore, if the PPLB condition can be satisfied, we should have *I* = −ε_HOMO_ and *A* = −ε_LUMO_.

Within the framework of ground-state DFT, a rigorous mapping between the other remaining KS/GKS orbital energies apart from the HOMO and LUMO and the QP energies has not been established. However, in practice the Koopmans-like relations have been proposed and adopted by many authors (Hill et al., 2000; Coropceanu et al., 2002; Vargas et al., 2005; Bartlett, 2009; Gritsenko and Baerends, 2009; Tsuneda et al., 2010; Dauth et al., 2011; Körzdörfer et al., 2012; Baerends et al., 2013; Bartlett and Ranasinghe, 2017; Puschnig et al., 2017; Ranasinghe et al., 2017; Thierbach et al., 2017). These relations have the form of $\varepsilon_{i}\approx -I_{i}^{\text{v}}=-\left[E_{i}\left(N-1\right)-E_{0}\left(N\right)\right]$ and $\varepsilon_{a}\approx -A_{a}^{\text{v}}=-\left[E_{0}\left(N\right)-E_{a}\left(N+1\right)\right]$. Here, the index *i* (*a*) denotes the occupied (virtual) KS/GKS orbital of the *N*-electron system from (to) which an electron is deprived (added), with $I_{i}^{\text{v}}$ ($A_{a}^{\text{v}}$) being the corresponding vertical IP (EA). It is easily recognized that these vertical IPs and EAs coincide with the energies of quasiholes and quasielectrons, i.e., $\omega_{i}=-I_{i}^{\text{v}}$ and $\omega_{a}=-A_{a}^{\text{v}}$, respectively. Computationally, approximating QP energies by KS/GKS orbital energies has the advantage of requiring only a single self-consistent field (SCF) calculation for the ground state of the interested molecule.

The excited-state properties of molecular systems can be probed by photon absorption experiments (Onida et al., 2002). However, theoretical characterization of the excited states is rather challenging because the excited electron and the resulting hole cannot be treated separately. Numerous methods have been developed for the calculation of excitation energies. Coupled cluster (CC) (Schreiber et al., 2008; Silva-Junior et al., 2008; Winter et al., 2013; Wang et al., 2014; Dreuw and Wormit, 2015; Jacquemin et al., 2015) and multi-reference methods (Andersson et al., 1990; Potts et al., 2001; Slavicek and Martinez, 2010; Hoyer et al., 2016) are able to describe electronic excited states with a high accuracy. However, the expensive computational cost makes the application of these methods to large systems rather difficult. As a straightforward extension of the *GW* approach (Hedin, 1965; Hybertsen and Louie, 1986; Onida et al., 2002), the Bethe–Salpeter equation (BSE) (Rohlfing and Louie, 2000; Onida et al., 2002; Jacquemin et al., 2017) provides another method for the calculation of excited states, which is however also quite expensive. The time-dependent DFT (TDDFT) (Runge and Gross, 1984; Casida, 1995) is in principle an exact extension of the ground-state DFT, and it has been widely employed to study neutral excitations. Despite its success, TDDFT faces several challenges, such as double excitation character, multi-reference problems, and high-spin excited states (Ipatov et al., 2009; Laurent and Jacquemin, 2013; Santoro and Jacquemin, 2016).

Recently, a simple method has been proposed, which attempts to acquire excitation energies by using only the KS/GKS orbital energies of the molecular cations calculated by ground-state DFT (Haiduke and Bartlett, 2018; Mei et al., 2019). Such a method is referred to as the QE-DFT (QP energies from DFT), which has been employed to describe excited-state potential energy surfaces and conical intersections (Mei and Yang, 2019). Following the idea of QE-DFT, molecular excitation energies have also been expressed by KS/GKS orbital energies obtained with long-range corrected functionals (Hirao et al., 2020). Details about QE-DFT are to be presented in section 2.1.

In addition to neutral molecules, in this work we also consider the resonance states of temporary anions. A temporary anion has an energy higher than that of the neutral species, and thus its EA has a negative value. This means the anion is unstable and lasts only a short time. Although temporary anions cannot be studied by traditional spectroscopic techniques, they can be observed via resonances (sharp variations) in the cross-sections of various electron scattering processes (Jordan and Burrow, 1987). In the gas phase, the resonances can be identified by the electron transmission spectroscopy (Sanche and Schulz, 1972; Schulz, 1973; Jordan and Burrow, 1987). Since these resonances belong to the continuous part of the spectrum, they cannot be addressed by conventional electronic structure methods for bound states. A number of theoretical methods have been proposed to tackle the problem of temporary anions. For instance, it has been proposed that the attractive components of electron-molecule interaction are combined with a long-range repulsive potential to produce a barrier, behind which the excess electron can be temporarily trapped (Jordan et al., 2014). Moreover, the negative EAs have been studied by the *GW* method (Hedin, 1965; Hybertsen and Louie, 1986; Govoni and Galli, 2018), the electron-propagator methods (Longo et al., 1995; Ortiz, 2013; Dolgounitcheva et al., 2016), and the equation-of-motion coupled cluster (EOM-CC) approach (Stanton and Bartlett, 1993; Nooijen and Bartlett, 1995; Dutta et al., 2014; Jagau et al., 2017; Skomorowski et al., 2018; Ma et al., 2020), again with considerable computational cost.

In order to describe the unbound resonance states within the DFT approach, Tozer and De Proft (2005) have proposed an approximate approach to evaluate the EA based on the KS frontier orbital energies (Kohn and Sham, 1965) and the accurate IP. Zhang et al. (2018) have used directly the negative of GKS eigenvalue of the neutral ground-state molecule as an approximation of EA corresponding to the resonance state of the anion. The good accuracy was made possible because of the use of the global scaling correction (GSC) (Zheng et al., 2011), which will be introduced later. At the same time, another method has been developed to evaluate the negative EA from the GKS eigenvalue of the neutral ground states (Carmona-Espíndola et al., 2020). Different from GSC, this method is designed to impose the derivative discontinuity of the exact XC potential. Our work (Zhang et al., 2018) proceeded the work of Mei et al. (2019) and that of Haiduke and Bartlett (Haiduke and Bartlett, 2018) in the direct use of GKS eigenvalues of the *N*-electron ground state to approximate the excited state energy of the corresponding (*N* + 1)-electron system, with the excited state of the (*N* + 1)-electron system being a unbound resonance state.

For achieving an accurate prediction of QP energies with ground-state density functional methods, it is crucial to reduce the delocalization error associated with the adopted DFA. Enormous efforts have been made, which have led to the development of the GSC (Zheng et al., 2011) and local scaling correction (LSC) (Li et al., 2015) approaches, which alleviate substantially the delocalization error of various DFAs for systems involving global and local fractional electron distributions, respectively. This is done by imposing explicitly the PPLB condition on the form of DFA. Recently, a localized orbital scaling correction (LOSC) (Li et al., 2017; Su et al., 2020) has been constructed to join the merits of GSC and LSC. The LOSC approach is capable of correcting the energy, energy derivative, and electron density of any finite system in a self-consistent and size-consistent manner. In particular, the LOSC approach has been applied in conjunction with the QE-DFT to predict QP and excitation energies of molecules (Mei et al., 2019).

In this work, we revisit the non-empirical GSC approach (Zheng et al., 2011, 2013, 2015; Zhang et al., 2015) and explore the possibility of using it to achieve an accurate prediction of QP and excitation energies. With a perturbative treatment of the orbital relaxation induced by the addition (removal) of an infinitesimal amount of electron to (from) a molecule, the GSC approach has been demonstrated to improve systematically the prediction of KS frontier orbital energies and band gaps of molecules (Zhang et al., 2015). Based on the idea of QE-DFT, we will extend the scope of GSC from the frontier orbitals to the other KS/GKS orbitals.

The remainder of this paper is organized as follows. In section 2, we present the QE-DFT method to calculate the QP and excitation energies within the framework of ground-state DFT, as well as the GSC approach to achieve the accurate KS/GKS orbital energies. In section 3, numerical results of the QP energies, electronic excitation energies, and resonance energies are presented and discussed. Finally, we summarize this work in section 4.

## 2. Methodology

### 2.1. QE-DFT Method for the Calculation of QP, Excitation, and Resonance Energies

In the QE-DFT method, the following Koopmans-like relations are adopted, which use the energies of occupied and virtual KS/GKS orbitals to approximate the quasihole and quasielectron energies, respectively.

(3) $$\begin{array}{l} \varepsilon_{a}\left(N\right)\approx \omega_{a}\left(N\right)=E_{a}\left(N+1\right)-E_{0}\left(N\right),\\ \;\varepsilon_{i}\left(N\right)\approx \omega_{i}\left(N\right)=E_{0}\left(N\right)-E_{i}\left(N-1\right). \end{array}$$

Here, {ε_*i*_(*N*)} and {ε_*a*_(*N*)} are the occupied and virtual orbital energies of the *N*-electron system, respectively. *E*_*a*_(*N* + 1) denotes the energy of the (*N* + 1)-electron system formed by adding an excess electron to the *a*th virtual orbital of the *N*-electron system at its ground state. Note that the subscript *a* refers to the *N*-electron system, and the value of such an orbital index may vary in the (*N* + 1)-electron system because of the relaxation and re-ordering of the orbitals upon the perturbation induced by electron addition. A similar argument applies to the *i*th occupied orbital of the *N*-electron system.

From Equation (3), it is obvious that the excitation energy of an electron from the HOMO to a virtual orbital of the *N*-electron system, which corresponds to the *a*th orbital of the (*N* − 1)-electron system, can be calculated as follows (Haiduke and Bartlett, 2018; Mei et al., 2019):

(4) $$\begin{array}{l} \Delta E_{a}\left(N\right)\equiv E_{a}\left(N\right)-E_{0}\left(N\right)\\ \;=\left[E_{a}\left(N\right)-E_{0}\left(N-1\right)\right]-\left[E_{0}\left(N\right)-E_{0}\left(N-1\right)\right]\\ \;=\omega_{a}\left(N-1\right)-\omega_{\text{LUMO}}\left(N-1\right)\\ \;\approx \varepsilon_{a}\left(N-1\right)-\varepsilon_{\text{LUMO}}\left(N-1\right). \end{array}$$

Here, in the second equality of Equation (4), we have chosen to use the (*N* − 1)-electron system as a reference system. This means the electronic excitation from the HOMO to a virtual orbital can be regarded as consisting of two steps: first an electron is removed from the HOMO of the *N*-electron system, giving rise to an (*N* − 1)-electron system in its ground state, and then an excess electron is put to the *a*th virtual orbital of the (*N* − 1)-electron system, which is energetically higher than the frontier orbitals, resulting in an excited *N*-electron system. Accordingly, *E*_0_(*N* − 1) is the ground-state energy of the (*N* − 1)-electron system, and *E*_*a*_(*N*) denotes the energy of the *N*-electron system that is finally obtained. Thus, Equation (4) can describe the excitation of an electron from the HOMO to any virtual KS/GKS orbital (LUMO and above), as long as the orbital finds its counterpart in the (*N* − 1)-electron system.

Specifically, if we presume the (*N* − 1)-electron reference system contains one more spin-α electrons than spin-β electrons, the first triplet-state excitation energy of the *N*-electron system is calculated by

(5) $$\begin{array}{l} \Delta E_{\text{T}1}\left(N\right)\equiv E_{\text{T}1}\left(N\right)-E_{0}\left(N\right)\approx \varepsilon_{\alpha ,\text{LUMO}}\left(N-1\right)-\varepsilon_{\beta ,\text{LUMO}}\left(N-1\right). \end{array}$$

Higher triplet-state excitation energies can be calculated similarly.

We now consider the first singlet excited state formed by adding a spin-β electron to the *a*th virtual orbital of the ground-state (*N* − 1)-electron system. It is well-known that the accurate calculation of open-shell singlet states is quite challenging for the density functional methods within the KS/GKS scheme. This is because the electronic wavefunction naturally involves more than one Slater determinant, and such a multireference character is hardly captured by the presently used DFAs due to their intrinsic static correlation error (Cohen et al., 2008b). Moreover, an open-shell singlet wavefunction in the form of a single Slater determinant of KS/GKS orbitals is not an eigenstate of the total spin operator. In practice, people have attempted to circumvent the problem of static correlation error by explicitly using more than one Slater determinant. For instance, the singlet-state energy of an *N*-electron system has been written as (Ess et al., 2011)

(6) $$\begin{array}{l} E_{\text{S}}\left(N\right)=E_{\text{M}}\left(N\right)+\chi \left[E_{\text{M}}\left(N\right)-E_{\text{T}}\left(N\right)\right]. \end{array}$$

Here, *E*_M_(*N*) represents the energy of a single-Slater-determinant wavefunction with the excited spin-β electron occupying the virtual orbital. The second term on the right-hand side is a correction to the singlet-state energy, which accounts for the spin contamination of the single-Slater-determinant wavefunction, with χ being a parameter. The singlet-state excitation energy of the *N*-electron is thus obtained as

(7) $$\begin{array}{l} \Delta E_{\text{S}}\left(N\right)\equiv E_{\text{S}}\left(N\right)-E_{0}\left(N\right)\\ \;=\left[E_{\text{M}}\left(N\right)-E_{0}\left(N\right)\right]+\chi \left[E_{\text{M}}\left(N\right)-E_{\text{T}}\left(N\right)\right]\\ \;\approx \left[\varepsilon_{\beta ,\text{LUMO}+a}\left(N-1\right)-\varepsilon_{\text{β},\text{LUMO}}\left(N-1\right)\right]\\ \;+\chi \left[\varepsilon_{\beta ,\text{LUMO}+a}\left(N-1\right)-\varepsilon_{\alpha ,\text{LUMO}+a}\left(N-1\right)\right]. \end{array}$$

To improve the accuracy of Δ*E*_S_, a spin purification procedure has been proposed (Ziegler et al., 1977), which amounts to χ = 1 in Equation (6). Specifically, the first singlet-state excitation energy is calculated by

(8) $$\begin{array}{r} \Delta E_{\text{S}1}\left(N\right)\equiv E_{\text{S}1}\left(N\right)-E_{0}\left(N\right)\approx 2\varepsilon_{\beta ,\text{LUMO}+1}\left(N-1\right)\\ -\varepsilon_{\alpha ,\text{LUMO}}\left(N-1\right)-\varepsilon_{\beta ,\text{LUMO}}\left(N-1\right). \end{array}$$

Obviously, with the QE-DFT method, the calculation of excitation energies requires the SCF calculations to be performed explicitly only for the ground-state (*N* − 1)-electron system.

Regarding temporary anions, we only consider the scenario that the LUMO of the neutral molecule is already an unbound orbital, which corresponds to a negative EA. Consequently, addition of an excess electron to the LUMO gives rise to a resonant state. Traditionally, the molecular EA is obtained by performing SCF calculations separately for the neutral and anionic systems and taking the energy difference between them. This is referred to the ΔSCF method. However, in practice it is extremely difficult to carry out an SCF calculation for the anionic species if it is in a resonant state.

Since the LUMO is a frontier orbital, the PPLB condition holds exactly, and thus the negative EA can be obtained directly from the positive LUMO energy via the following equality:

(9) $$\begin{array}{l} A=-\varepsilon_{\text{LUMO}}\left(N\right). \end{array}$$

By using Equation (9), the SCF calculation on the temporary anion that is potentially problematic is no longer needed.

### 2.2. GSC Approach for the Accurate Prediction of KS/GKS Orbital Energies

From section 2.1, the prediction of QP and excitation energies is transformed to the accurate calculation of KS/GKS orbital energies. To this end, we employ a non-empirical GSC approach developed in our previous works Zheng et al. (2013); Zhang et al. (2015) to reduce the delocalization error of some frequently used DFAs. It has been demonstrated that the GSC approach greatly improves the accuracy of the frontier KS/GKS orbitals. In the following, we shall go beyond the frontier orbitals and extend the application of GSC to all the KS/GKS orbitals.

In the KS or GKS scheme, the total electronic energy in the ground state is *E*_0_(*N*) = *T*_s_ + *V*_ext_ + *J* + *E*_xc_. With the KS/GKS orbitals fixed as the electron number is varied, the KS kinetic energy *T*_s_ and external potential energy *V*_ext_ are linear in ρ(**r**), while the electron Coulomb energy *J* is quadratic, and *E*_xc_ is usually non-linear in ρ(**r**). The GSC approach establishes a linear energy function that satisfies the PPLB condition,

(10) $$\begin{array}{l} {\tilde{E}}_{0}\left(N+n\right)\equiv \left(1-n\right)E_{0}\left(N\right)+nE_{0}\left(N+1\right), \end{array}$$

by linearizing both *J* and *E*_xc_ with respect to the fractional electron number *n*. The difference between Ẽ_0_ and *E*_0_ is just the GSC for the energy:

(11) $$\begin{array}{l} \Delta E_{0}^{\text{GSC}}={\tilde{E}}_{0}\left(N+n\right)-E_{0}\left(N+n\right). \end{array}$$

Here, $\Delta E_{0}^{\text{GSC}}$ can express explicitly by the electron density $\rho \left(\text{r}\right)=\underset{m}{\sum }n_{m}{\left[\phi_{m}\left(\text{r}\right)\right]}^{2}$ and some other quantities, where ϕ_*m*_(**r**) and *n*_*m*_ are the *m*th KS/GKS orbital and electron occupation number, respectively. For simplicity, the spin indices are omitted.

The addition of the *n* fractional electron to the LUMO presents a perturbation to the *N*-electron system, and the change in electron density in response to such a perturbation is

(12) $$\begin{array}{l} \delta \rho \left(\text{r}\right)=\rho^{N+n}\left(\text{r}\right)-\rho^{N}\left(\text{r}\right)=nf\left(\text{r}\right)+n^{2}\gamma \left(\text{r}\right)+\cdots \;, \end{array}$$

where $f\left(\text{r}\right)\equiv \underset{n\to 0}{\lim}\frac{\partial \rho \left(\text{r}\right)}{\partial n}{|}_{v_{\text{ext}}}$ and $\gamma \left(\text{r}\right)\equiv \underset{n\to 0}{\lim}\frac{1}{2}\frac{\partial^{2}\rho \left(\text{r}\right)}{\partial n^{2}}{|}_{v_{\text{ext}}}$ are the first- and second-order Fukui functions (Parr and Yang, 1984; Yang et al., 1984; Yang and Parr, 1985), respectively. Accordingly, the relaxation of KS/GKS orbitals upon the addition of *n* fractional electron can be expanded in a perturbative series as $\delta \phi_{m}\left(\text{r}\right)=\phi_{m}^{N+n}\left(\text{r}\right)-\phi_{m}^{N}\left(\text{r}\right)=n\delta \phi_{m}^{\left(1\right)}\left(\text{r}\right)+n^{2}\delta \phi_{m}^{\left(2\right)}\left(\text{r}\right)+\cdots \;$, with $\delta \phi_{m}^{\left(k\right)}\left(\text{r}\right)$ being the *k*th-order orbital relaxation. Thus, the Fukui functions can be expressed explicitly in terms of orbital relaxation. For instance, the first-order Fukui function is

(13) $$\begin{array}{l} f\left(\text{r}\right)=|\phi_{f}\left(\text{r}\right){|}^{2}+2\underset{m}{\sum }n_{m}\delta \phi_{m}^{\left(1\right)}\left(\text{r}\right)\phi_{m}\left(\text{r}\right). \end{array}$$

Here, the subscript *f* denotes the frontier orbital, with *f* = LUMO (*f* = HOMO) in the case of electron addition (removal). The explicit forms of orbital relaxation up to the third order have been derived and provided in Zhang et al. (2015), with all the perturbation Hamiltonian matrices determined by a self-consistent process. Ultimately, all orders of orbital relaxation and Fukui quantities are expressed in terms of {ϕ_*m*_(**r**)} and {ε_*m*_} of the *N*-electron system. The scaling correction to the frontier orbital energy is then evaluated by the Janak's theorem (Janak, 1978) in a post-SCF manner,

(14) $$\begin{array}{l} \Delta \varepsilon_{f}^{\text{GSC}}=\frac{\partial \Delta E_{0}^{\text{GSC}}}{\partial n}=\Delta \varepsilon_{f}^{\left(1\right)}+\Delta \varepsilon_{f}^{\left(2\right)}+\cdots \;, \end{array}$$

where $\Delta \varepsilon_{f}^{\left(k\right)}$ is the *k*th-order correction to the frontier orbital energy.

An accurate prediction of molecular IP and EA has been achieved by employing the GSC approach (Zheng et al., 2013, 2015; Zhang et al., 2015, 2018) via

(15) $$\begin{array}{l} I=-\varepsilon_{\text{HOMO}}^{\text{GSC}-\text{DFA}}=-\left(\varepsilon_{\text{HOMO}}^{\text{DFA}}+\Delta \varepsilon_{\text{HOMO}}^{\text{GSC}}\right), \end{array}$$

(16) $$\begin{array}{l} A=-\varepsilon_{\text{LUMO}}^{\text{GSC}-\text{DFA}}=-\left(\varepsilon_{\text{LUMO}}^{\text{DFA}}+\Delta \varepsilon_{\text{LUMO}}^{\text{GSC}}\right). \end{array}$$

In practical calculations, the perturbative series needs to be truncated at a certain order. It is worth pointing out that the accuracy of the GSC does not necessarily increase with further inclusion of higher order orbital relaxation. This is because the present form of GSC only treats the exchange energy *E*_x_, while the correlation energy *E*_c_ is presumed to be much smaller and hence its correction is omitted. However, the correction to *E*_c_ may have a comparable magnitude to the high-order corrections to *E*_x_. For instance, regarding the prediction of EA, while the inclusion of first-order orbital relaxation is found optimal for the LDA and GGA (such as BLYP, Becke, 1988; Lee et al., 1988), the inclusion of orbital relaxation up to second-order is most favorable for the hybrid functional B3LYP (Lee et al., 1988; Becke, 1993).

To extend the GSC approach beyond the frontier KS/GKS orbitals, we presume that the PPLB condition could be generalized to the following energy linearity relation:

(17) $$\begin{array}{l} {\tilde{E}}_{a}\left(N+n\right)\equiv \left(1-n\right)E_{0}\left(N\right)+nE_{a}\left(N+1\right). \end{array}$$

The GSC to the energy of the (*N* + *n*)-electron system is

(18) $$\begin{array}{l} \Delta E_{a}^{\text{GSC}}={\tilde{E}}_{a}\left(N+n\right)-E_{a}\left(N+n\right), \end{array}$$

where *E*_*a*_(*N* + *n*) is the energy of the (*N* + *n*)-electron system in an excited state, since the *n* fractional electron is now added to the *a*th virtual orbital of the *N*-electron system. Similarly, the changes of electron density and KS/GKS orbitals in response to the perturbation caused by the electron addition process, as well as their contributions to $\Delta E_{a}^{\text{GSC}}$, are calculated by using the self-consistent perturbation theory presented in Zhang et al. (2015). This finally gives rise to the GSC to the KS/GKS orbital energies:

(19) $$\begin{array}{l} \Delta \varepsilon_{a}^{\text{GSC}}=\frac{\partial \Delta E_{a}^{\text{GSC}}}{\partial n}=\Delta \varepsilon_{a}^{\left(1\right)}+\Delta \varepsilon_{a}^{\left(2\right)}+\cdots \;. \end{array}$$

Likewise, for the scenario that *n* fractional electron is deprived from the *i*th occupied orbital of the *N*-electron system, we have

(20) $$\begin{array}{l} \Delta \varepsilon_{i}^{\text{GSC}}=\frac{\partial \Delta E_{i}^{\text{GSC}}}{\partial n}=\Delta \varepsilon_{i}^{\left(1\right)}+\Delta \varepsilon_{i}^{\left(2\right)}+\cdots \;. \end{array}$$

With the QE-DFT method, we can now use the scaling corrected KS/GKS orbital energies to approximate the QP energies and the related vertical IPs and EAs as follows:

(21) $$\begin{array}{l} \omega_{i}=-I_{i}^{\text{v}}\approx \varepsilon_{i}^{\text{GSC}-\text{DFA}}=\varepsilon_{i}^{\text{DFA}}+\Delta \varepsilon_{i}^{\text{GSC}},\\ \omega_{a}=-A_{a}^{\text{v}}\approx \varepsilon_{a}^{\text{GSC}-\text{DFA}}=\varepsilon_{a}^{\text{DFA}}+\Delta \varepsilon_{a}^{\text{GSC}}. \end{array}$$

## 3. Results and Discussions

### 3.1. QP Energies

#### 3.1.1. Quasihole Energies of Molecules

Because of the lack of highly accurate experimental or theoretical data for the molecular quasielectron energies (except for those associated with the LUMOs), in this work we only compare the calculated quasihole energies that are associated with the occupied KS/GKS orbitals to the reference data available in the literature.

We first look into 56 quasihole energies of 12 molecules by calculating the scaling corrected orbital energies, and make comparison with experimentally measured vertical IPs. The examined molecules are cyanogen, CO, acetylene, water, ethylene, ammonia, acetonitrile, fluoromethane, benzene, naphthalene, furan, O2, and formic acid, which exhibit diversified geometric and electronic features. Among these molecules, the geometries of benzene and naphthalene are extracted from Mei et al. (2019), while the structures of the other molecules are optimized with the B3LYP/6-311+g** method by using the Gaussian09 package (Frisch et al., 2009).

The GSC approach presented in section 2.2 is employed to correct the orbital energies calculated by various mainstream DFAs, including the LDA, the GGAs (BLYP and PBE, Perdew et al., 1996), and the hybrid functional B3LYP. For these DFAs, the orbital relaxation up to the second order is considered for calculating the scaling corrections of the occupied orbital energies. The GSC approach is implemented in an in-house built quantum chemistry software package QM^4^D (Hu et al., 2020).

**Figure 1:**
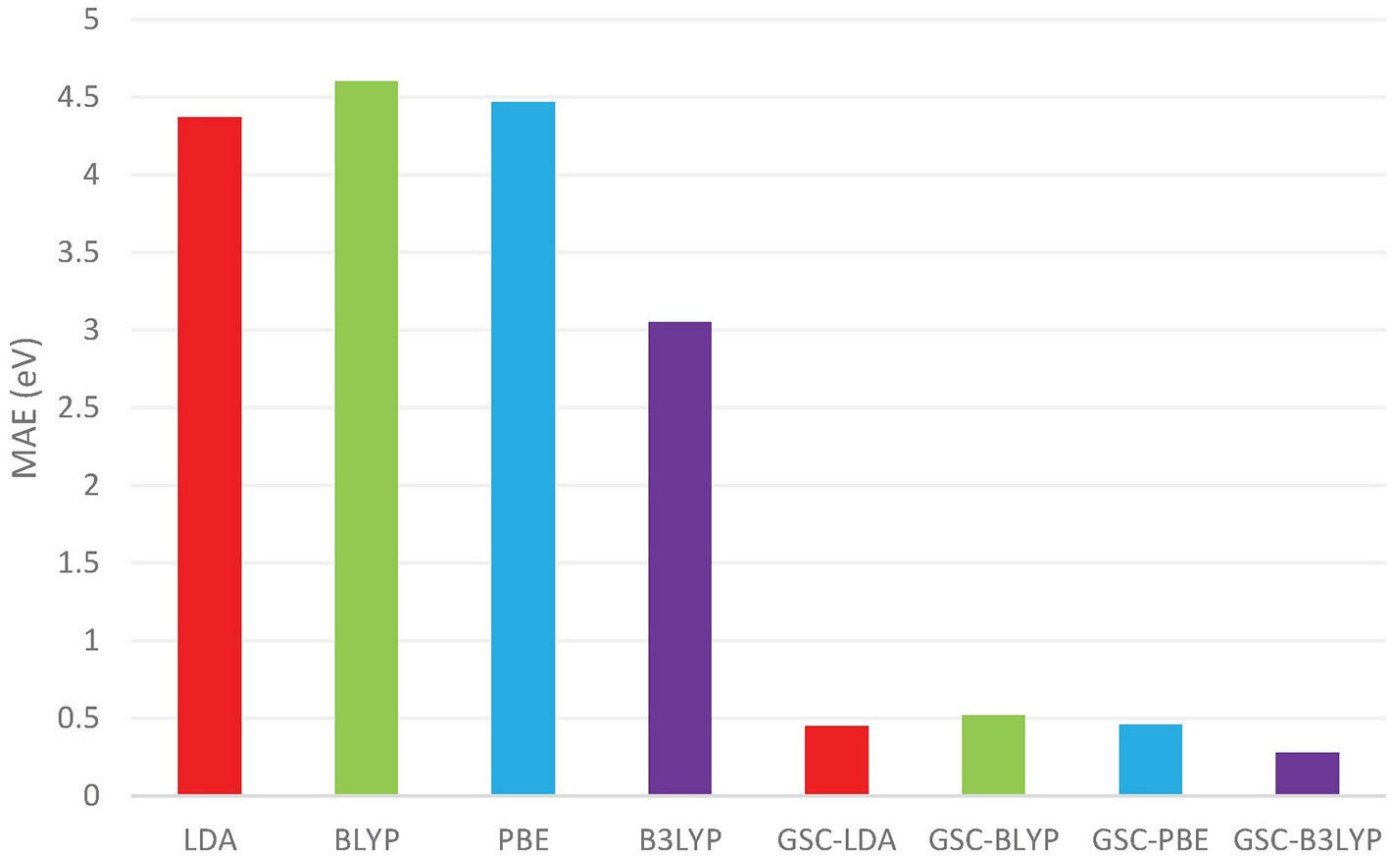
The mean absolute errors (MAEs) (in units of eV) between the occupied Kohn–Sham (KS)/generalized KS (GKS) orbital energies {ε_*i*_} calculated by various density functional approximations (DFAs) and the experimentally measured quasihole energies {ω_*i*_}. The experimental data are extracted from Chong et al. (2002) and Schmidt (1977). The basis set adopted in the density functional calculations is aug-cc-pVTZ (Kendall et al., 1992; Woon and Dunning Jr, 1993).

Figure 1 compares the averaged deviations of the calculated $\left\{\varepsilon_{i}^{\text{DFA}}\right\}$ and $\left\{\varepsilon_{i}^{\text{GSC}-\text{DFA}}\right\}$ from the quasihole energies {ω_*i*_} extracted from the experimentally measured vertical IPs. It is shown clearly that the mean absolute errors (MAEs) associated with the original DFAs are as large as several eVs, while by applying the GSC approach, the MAEs are substantially reduced to less than 0.5 eV. Take the B3LYP functional as an example. It yields an MAE of 3.05 eV, which is the smallest among all the uncorrected DFAs, and the MAE is greatly reduced to 0.28 eV by using the GSC-B3LYP. If instead the orbital relaxation is treated up to the first and third order, the MAE becomes 0.74 eV and 0.43 eV, respectively. The dependence on the order of orbital relaxation is consistent with the trend observed in our previous work (Zhang et al., 2015).

In a previous study by Chong et al. (2002), 10 out of 12 molecules examined in Figure 1 (without benzene and naphthalene) have been investigated by calculating their KS/GKS orbital energies by using an approximate XC potential obtained with the statistical averaging of (model) orbital potentials (SAOP). For these 10 molecules, the MAE reported in Chong et al. (2002) is 0.38 eV, while the GSC-B3LYP yields a somewhat smaller MAE of 0.28 eV, albeit the different molecular geometries and basis sets adopted.

**Figure 2:**
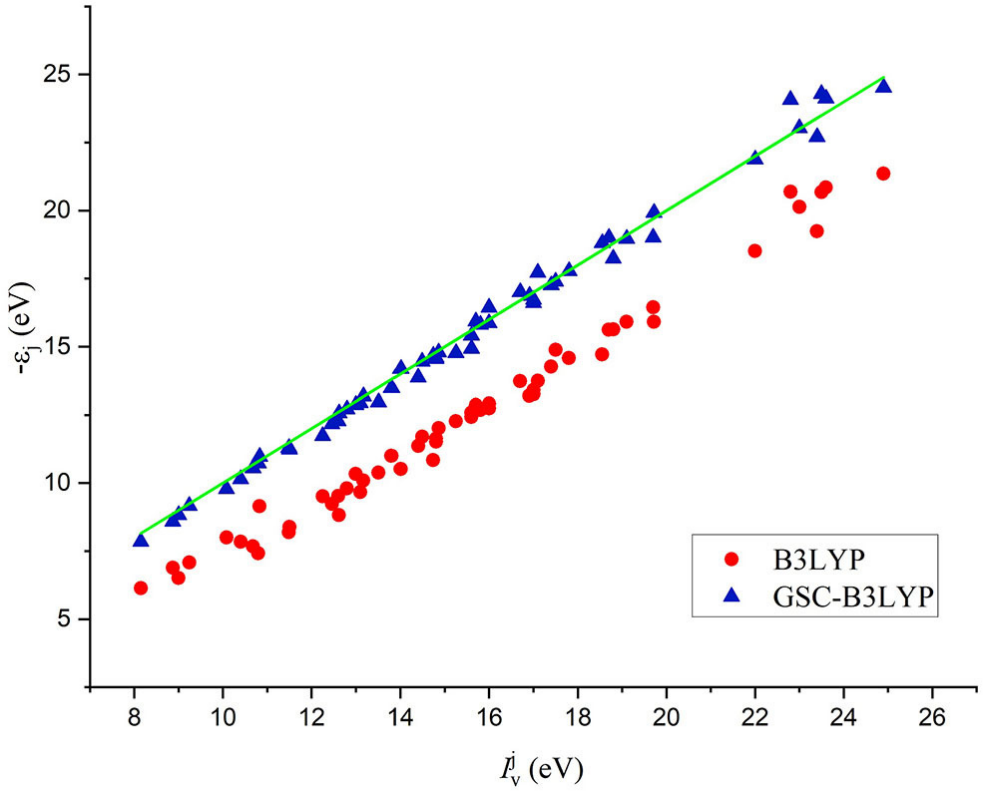
A comparison between 56 Kohn–Sham (KS)/generalized KS (GKS) orbital energies {−ε_*i*_} calculated by B3LYP and GSC-B3LYP and the corresponding vertical ionization potentials (IPs) $\left\{I_{i}^{\text{v}}\right\}$ measured experimentally for 12 molecules (see the main text). The green solid line indicates exact equality.

The comparison between the individual orbital energies {−ε_*i*_} calculated by B3LYP and GSC-B3LYP and the experimentally measured vertical IPs $\left\{I_{i}^{\text{v}}\right\}$ is depicted in Figure 2. It is apparent that the uncorrected orbital energies deviate systematically and significantly from the experimental QP energies, while such deviations are largely alleviated by applying the GSC approach.

#### 3.1.2. Photoemission Spectra

The QP energies can also be extracted from the peak positions of experimentally measured photoemission spectra (PES). We employ the QE-DFT to study the PES of 14 molecules. The same molecular geometries and basis set (cc-pVTZ, Dunning, 1989; Woon and Dunning Jr, 1993) as those adopted in Mei et al. (2019) are used here. The PES are simulated by setting the energy of each KS/GKS orbital as the center of a QP peak, and assuming all QP peaks have the same amplitude and are broadened by the same Gaussian function $e^{-{\left(\varepsilon -\varepsilon_{i}\right)}^{2}/2\lambda^{2}}$ with λ = 0.2eV.

**Figure 3:**
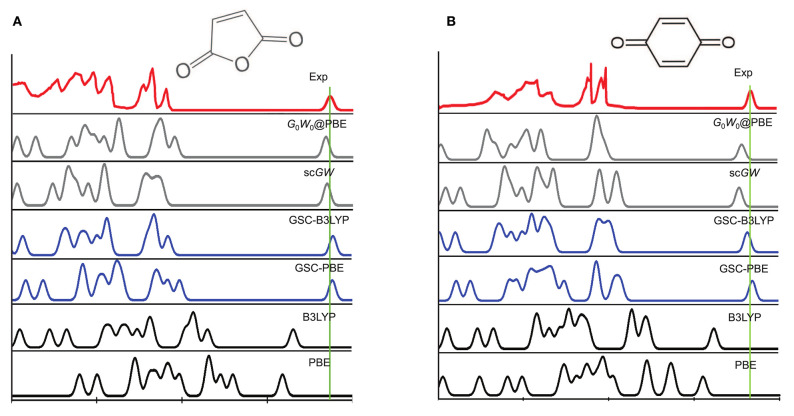
A comparison between the experimental and simulated photoemission spectra (PES) of **(A)** a maleic anhydride and **(B)** a benzoquinone. The experimental PES are extracted from Brundle et al. (1972), Knight et al. (2016), with the rightmost quasielectron peak added manually. The quasielectron peak is centered at the experimental EA of the molecule, and is broadened artificially by a Gaussian function with the width of λ = 0.2 eV. The simulated PES by using the QE-DFT is explained in the main text, and the results of the self-consistent *GW* (sc*GW*) and non-self-consistent *G*_0_*W*_0_ methods are extracted from Knight et al. (2016).

Figure 3 depicts the experimentally measured and theoretically simulated PES of a maleic anhydride and a benzoquinone, while those of the other 12 molecules are presented in Supplementary Material. Clearly, both the PBE and B3LYP yield considerable errors in the peak positions of the simulated PES. More specifically, they tend to predict much too high quasihole energies and too low quasielectron energies. This is because the uncorrected DFAs (PBE and B3LYP) suffer from delocalization error, as they violate the rigorous PPLB condition and the extended energy linearity relation.

The use of GSC improves significantly the simulated PES. For GSC-PBE, the orbital relaxation is considered up to the first and second order for the virtual and occupied KS/GKS orbitals, respectively; while for GSC-B3LYP, the orbital relaxation is included up to second order for all the KS/GKS orbitals. From the comparison shown in Figure 3, it is evident that the GSC-DFAs achieve at least the same level of accuracy as the results of *GW* method (Knight et al., 2016). Moreover, the computational cost of the QE-DFT method by using a GSC-DFA is supposedly much cheaper than that of the *GW* method, because the former requires only a single SCF calculation at the DFT level.

### 3.2. Energies of Low-Lying Excited States

We now turn to the energies of low-lying excited states of molecules. By employing the QE-DFT method, we carry out calculations on 48 low excitation energies of the 16 molecules investigated previously in Mei et al. (2019). The cationic species of all these molecules indeed contain one more spin-α electrons than spin-β electrons, and hence their triplet and singlet excitation energies are computed by using equations 5 and 7, respectively. Since the calculations involve only the virtual KS/GKS orbitals of the cations, the orbital relaxation is considered up to the second order for GSC-B3LYP and up to the first order for other GSC-DFAs, respectively.

**Figure 4:**
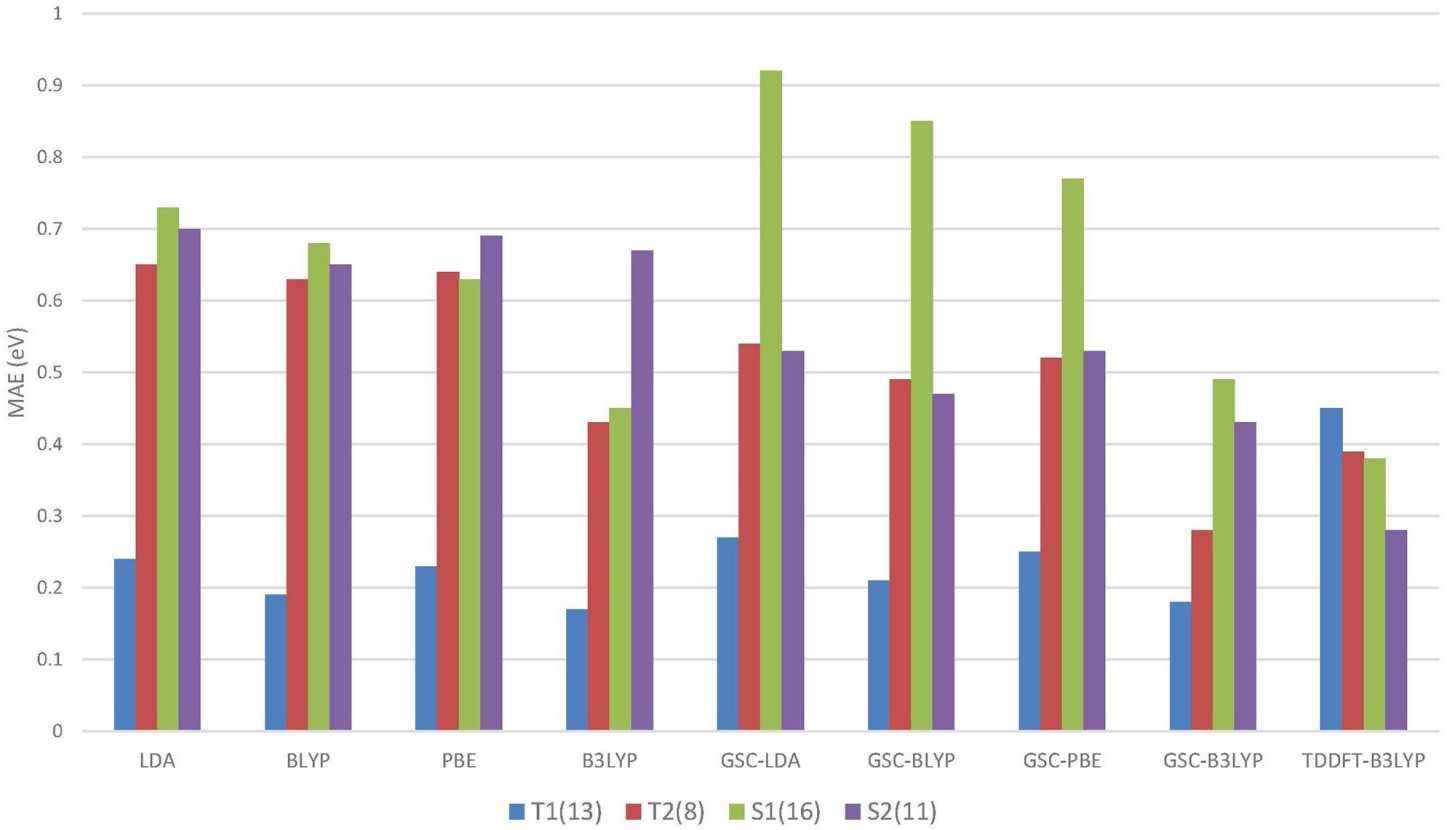
The mean absolute errors (MAEs) of the energies of different types of excitations calculated by using the QE-DFT method with various density functional approximations (DFAs) and the basis set 6-311++G(3df, 3pd). For comparison purpose, the MAEs of the TDDFT-B3LYP results extracted from Mei et al. (2019) are also displayed. Excitation energies calculated by high-level wavefunction methods are used as the reference data (Schreiber et al., 2008). T1 and S1 (T2 and S2) refer to the triplet and singlet HOMO to LUMO (LUMO+1) excitations, respectively. The numbers in the parentheses record the numbers of energy data belonging to the different types of excitations.

Figure 4 compares the MAEs of different types of excitation energies calculated by various DFAs, and the detailed results can be found in the Supplementary Material.

Intriguingly, for the lowest (HOMO-to-LUMO) triplet excitations, the uncorrected DFAs yield reasonably accurate excitation energies, and the application of the GSC approach does not lead to any improvement. In particular, B3LYP yields an MAE as small as 0.17 eV for the T1 excitations. Such an appealing accuracy is likely due to the cancellation of delocalization error. Equation (5) involves the difference between a pair of virtual orbital energies. Thus, when two virtual orbitals are close in energy, their associated delocalization errors are expected to cancel out (Mei et al., 2019). Consequently, the GSC approach does not help. Such an error cancellation mechanism becomes less favorable for higher excitations. For instance, as displayed in Figure 4, the uncorrected DFAs tend to yield a larger MAE for the T2 excitations, and applying the GSC indeed leads to improved accuracy. The latter is because the scaling correction to each individual QP energy starts to take effect.

For the S1 excitations, the GSC-DFAs yield MAEs that are somewhat larger than the original DFAs. This is because a second type of systematic error of the DFAs, the static correlation error, becomes prominent and significantly affects the calculated Δ*E*_S1_. Taking the LDA functional as an example. If the first singlet excited state is described by a single Slater determinant, i.e., by setting χ = 0 in Equation (6) and (7), the MAEs of Δ*E*_S1_ are 1.44eV and 1.33eV for the original LDA and GSC-LDA, respectively. In contrast, after adopting the spin purification procedure (by setting χ = 1), the MAEs reduce to 0.73eV and 0.92eV for the original LDA and GSC-LDA, respectively. Therefore, the spin purification procedure indeed diminishes the MAE of Δ*E*_S1_ by circumventing the problem of static correlation error. However, the somewhat larger MAE of the GSC-LDA seems to indicate that the spin purification formula is not entirely compatible with the present GSC scheme. For higher singlet excitations, the static correlation error becomes much less significant, as signified by the much smaller second term on the right-hand side of Equation (6). Consequently, the MAE of Δ*E*_S2_ experiences a rather minor change by invoking the spin purification. For instance, the MAE increases slightly from 0.68eV to 0.70eV with the original LDA, while it reduces slightly from 0.57eV to 0.53eV with the GSC-LDA.

Among all the DFAs examined, the GSC-B3LYP functional achieves an optimal performance for all the low-lying excitations studied. The overall accuracy of GSC-B3LYP is comparable to the TDDFT-B3LYP. This affirms that it is entirely possible and practical to access excited-state properties of molecules within the framework of ground-state DFT.

We further extend our test to cover three transition metal atoms M (M = Cu, Ag, Au) and nine transition metal compounds MX (X = F, Cl, Br). The calculated results are presented in Table 1 along with the CCSD(T) results and the available experimental data. The small MAEs between the calculated results and experimental data further affirm the applicability of the GSC approach.

**Table 1:** Calculated and experimental ionization potentials (IPs) and T1 excitation energies of various transition metal atoms and compounds.

		**Final state**	**Exp**.	**CCSD(T)**	**B3LYP**	**GSC-B3LYP**
IP	Cu	d^10^	7.73	7.69	5.35	7.90
	Ag	d^10^	7.58	7.52	5.30	7.80
	Au	d^10^	9.23	9.13	6.61	9.28
MAE^*a*^				0.07	2.43	0.14
T1	Cu	d^10^p^1^	3.81	3.87	4.92	3.94
	Ag	d^10^p^1^	3.84	3.74	4.80	3.87
	Au	d^10^p^1^	4.95	5.01	6.06	5.00
	CuF	^3^Σ^+^	1.81	1.81	2.73	2.05
	CuCl	^3^Σ^+^	2.35	2.43	3.24	2.35
	CuBr	^3^Σ^+^	2.54	2.56	3.19	2.35
	AgF	1^3^Σ^+^	3.09	3.27	3.26	2.54
	AgCl	1^3^Σ^+^	N/A	3.50	3.66	2.97
	AgBr	1^3^Σ^+^	N/A	3.37	3.43	2.83
	AuF	1^3^Σ^+^	N/A	2.08	2.17	1.80
	AuCl	1^3^Σ^+^	N/A	2.53	2.61	2.14
	AuBr	1^3^Σ^+^	N/A	2.60	2.52	2.09
MAE^*a*^				0.07	0.83	0.17
MAE^*b*^					0.50	0.31

*The experimental excitation energies and the CCSD(T) calculation results are extracted from Guichemerre et al. (2002). The experimental geometries (Guichemerre et al., 2002) and the def2-TZVPD basis set (Schäfer et al., 1994; Rappoport and Furche, 2010) are used for all the calculations*.

a *The MAE is between the calculated values and the experimental data*.

b *The MAE is between the DFT and the CCSD(T) results*.

### 3.3. Resonance Energies of Temporary Anions

For a temporary anion in a resonant state, the corresponding neutral molecule has a negative EA, for which the conventional ΔSCF method often yields problematic results. This is because the choice of an appropriate basis set is difficult for the SCF calculation of a temporary anion. On the one hand, the energy of a temporary anion is rather sensitive to the inclusion of diffuse basis functions (Guerra, 1990). On the other hand, the diffuse basis functions may artificially delocalize the excess electron (Cohen et al., 2008c, 2012), and thus result in incorrect electron density distribution.

Alternatively, using the scaling corrected LUMO energy to determine the energy of the temporary anion has made impressive progress. It has been demonstrated that the GSC-PBE functional predicts highly accurate negative EAs by using Equation (16) (Zhang et al., 2018). For a set of 38 molecules proposed in Tozer and De Proft (2005), the resulting MAE is as small as 0.18 eV with the aug-cc-pVTZ basis set. Recently, a similar accuracy has been reached by the explicit inclusion of derivative discontinuity in the GGA exchange potential (Carmona-Espíndola et al., 2020). In this section, we extend our calculation to 26 new molecules that are beyond the above mentioned works, and hence expand the test set to a total of 64 molecules. The molecular geometries are optimized at the B3LYP/6-311+G** level with the Gaussian09 suite of programs (Frisch et al., 2009). For the GSC approach, the relaxation of KS/GKS orbitals is considered up to second-order for GSC-B3LYP, and to first-order for other GSC-DFAs, respectively.

Table 2 lists the experimental and calculated EAs of the newly added 26 molecules. The experimental data are extracted from Jordan and Burrow (1978), Chiu et al. (1979), and Ng et al. (1983), while the theoretical data take either the values of −ε_LUMO_ (or −ε_*a*_ if it is the *a*th virtual orbital that is related to the resonant state) or the energy difference between the neutral and anionic species (the ΔSCF method). More details are given in the Supplementary Material.

**Table 2:** Experimental and calculated electron affinities (EAs) of 26 new molecules that are not included in Tozer and De Proft (2005) and Zhang et al. (2018).

**Molecule**	**Exp**.	**LDA**	**BLYP**	**B3LYP**	**PBE**	**GSC- LDA**	**GSC- BLYP**	**GSC- B3LYP**	**GSC- PBE**	**ΔSCF- PBE**	**ΔSCF- B3LYP**
Monofluoroethylene	−1.91	1.25	0.95	0.31	1.03	−1.97	−2.18	−2.06	−2.15	−0.47	−0.52
*trans*-1,2-difluoroethylene	−1.84	1.32	1.01	0.39	1.07	−1.95	−2.18	−2.09	−2.16	−0.50	−0.58
*cis*-1,2-difluoroethylene^a^	−2.18	1.14	0.82	0.19	0.89	−2.18	−2.42	−2.34	−2.40	−0.36	−0.40
1,1-Difluoroethylene^a^	−2.39	1.09	0.79	0.17	0.84	−2.15	−2.32	−2.09	−2.33	−0.42	−0.47
Trifluoroethylene^a^	−2.45	1.06	0.74	0.11	0.77	−2.30	−2.53	−2.38	−2.53	−0.41	−0.45
Tetrafluoroethylene^b^	−3.00	0.82	0.47	0.21	0.49	−2.71	−3.04	−3.07	−3.03	−0.89	−0.91
Nitrogen	−2.20	2.18	1.88	0.98	1.92	−2.20	−2.41	−2.40	−2.41	N/A	−1.83
Formaldehyde	−0.86	2.91	2.57	1.75	2.66	−0.92	−1.18	−1.14	−1.14	N/A	−0.46
Butadiene	−0.62	2.12	1.76	1.18	1.90	−0.61	−0.93	−0.85	−0.82	N/A	−0.73
Biphenyl	−0.30	2.04	1.63	1.13	1.80	0.01	−0.40	−0.39	−0.24	N/A	−0.37
Trichloromethane	−0.35	2.47	2.22	1.55	2.31	−0.24	−0.44	−0.46	−0.38	−0.15	−0.26
Dichlorofluoromethane	−0.96	1.99	1.74	1.08	1.81	−0.90	−1.06	−1.00	−1.04	−0.36	−0.44
Dichlorodifluoromethane	−0.98	2.37	2.11	1.43	2.17	−0.61	−0.80	−0.79	−0.77	−0.42	−0.48
Dichloromethane	−1.23	1.74	1.52	0.90	1.59	−1.02	−1.08	−0.89	−1.09	−0.31	−0.38
Benzene	−1.15	1.44	1.06	0.50	1.21	−1.18	−1.52	−1.48	−1.40	−0.36	−0.42
CO	−1.80	2.24	1.94	1.12	2.00	−1.89	−2.07	−1.96	−2.08	−1.05	−1.11
Cyanogen	−0.58	3.87	3.48	2.84	3.61	−0.48	0.12	0.23	0.23	0.21	0.29
Propyne^d^	−2.95	0.13	0.02	−0.40	0.01	−2.29	−1.83	−1.23	−2.13	−0.40	−0.47
Butadiyne	−1.00	2.09	1.73	1.16	1.87	−0.77	−1.07	−0.96	−0.97	−0.25	−0.36
Tetramethylethylene^e^	−2.27	0.42	0.20	−0.31	0.28	−1.81	−1.79	−1.48	−1.86	−0.34	−0.41
Acetylene^c^	−2.60	0.57	0.32	−0.28	0.39	−2.51	−2.58	−2.36	−2.61	−0.46	−0.53
Acrylonitrile	−0.21	3.00	2.62	2.01	2.76	−0.01	−0.35	−0.27	−0.24	0.02	−0.16
1,4-Cyclohexadiene^a^	−1.75	1.05	0.67	0.34	0.81	−1.45	−1.80	−1.89	−1.68	−0.34	−0.56
Toluene	−1.11	1.39	1.01	0.46	1.16	−1.11	−1.45	−1.43	−1.33	−0.34	−0.42
Ethylbenzene	−1.17	1.37	0.99	0.47	1.14	−1.07	−1.43	−0.90	−1.31	−0.28	−0.37
Isopropylbenzene	−1.08	1.39	0.99	0.44	1.16	−1.01	−1.38	−1.41	−1.25	−0.26	−0.34
MAE		3.17	2.85	2.23	2.95	0.17	0.25	0.30	0.21	1.22	1.01

*The calculated EAs are obtained by using the ΔSCF method, or take the values of the uncorrected $-\varepsilon_{\text{LUMO}}^{\text{DFA}}$ or scaling corrected $-\varepsilon_{\text{LUMO}}^{\text{GSC}-\text{DFA}}$. All energies are in units of eV. The aug-cc-pVTZ basis set is adopted for all the calculated data listed in this table*.

a *The −ε_LUMO+1_ calculated with the GSC-B3LYP is taken as the EA of this molecule*.

b *The −ε_LUMO+1_ calculated with the GSC-B3LYP, GSC-BLYP, and GSC-PBE are taken as the EA of this molecule*.

c *The −ε_LUMO+2_ calculated with the GSC-B3LYP is taken as the EA of this molecule*.

d *The −ε_LUMO+2_ calculated with the GSC-B3LYP and −ε_LUMO+1_ calculated with other DFAs are taken as the EA of this molecule*.

e *The −ε_LUMO+3_ calculated with the GSC-B3LYP and −ε_LUMO+1_ calculated with other DFAs are taken as the EA of this molecule*.

**Figure 5:**
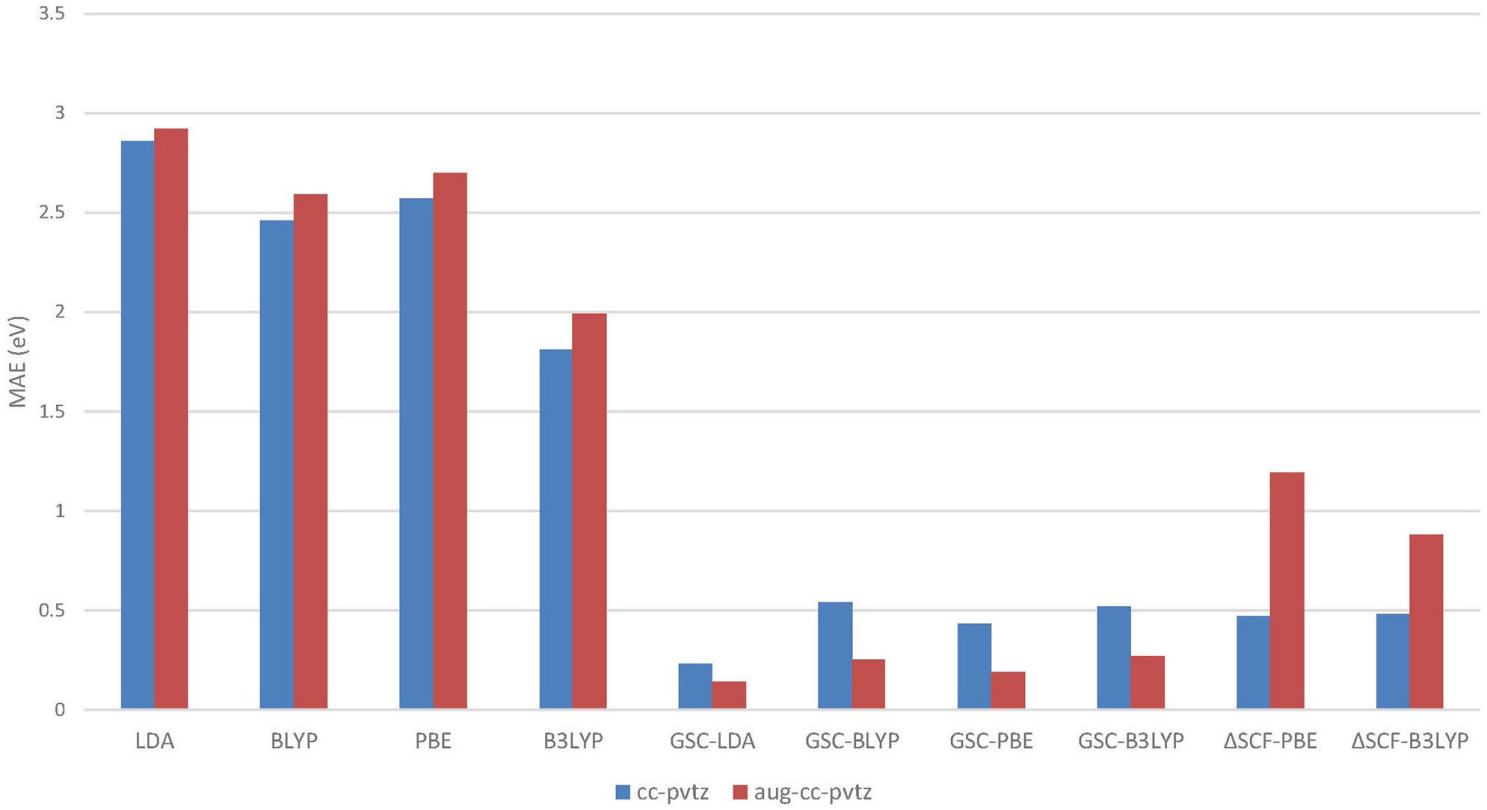
The mean absolute errors (MAEs) of the EAs for an extended set of 64 molecules calculated by employing the QE-DFT method with various density functional approximations (DFAs) and by using the ΔSCF method. Note that when the more diffuse aug-cc-pVTZ basis set is adopted, the energy of a certain virtual Kohn–Sham (KS)/generalized KS (GKS) orbital should be taken as the predicted EA; see Table 2 for details. If the orbital relaxation is considered up to the first order for the GSC-B3LYP, the MAEs become 0.21 and 0.28 eV with the cc-pVTZ and aug-cc-pVTZ basis sets, respectively.

Figure 5 visualizes the MAEs of the calculated EAs of the extended set of 64 molecules. Obviously, the application of the GSC approach greatly improves the accuracy of the virtual orbital energies (particularly the ε_LUMO_). The MAE is reduced from several eVs with the original DFAs to less than 0.5 eV with the GSC-DFAs. Moreover, the MAE is further reduced by adopting a more diffuse basis set. This is because a more complete basis set is more favorable for a perturbative treatment of scaling correction and orbital relaxation. The lowest MAE reached for the whole extended set is 0.14 eV with the GSC-LDA.

**Figure 6:**
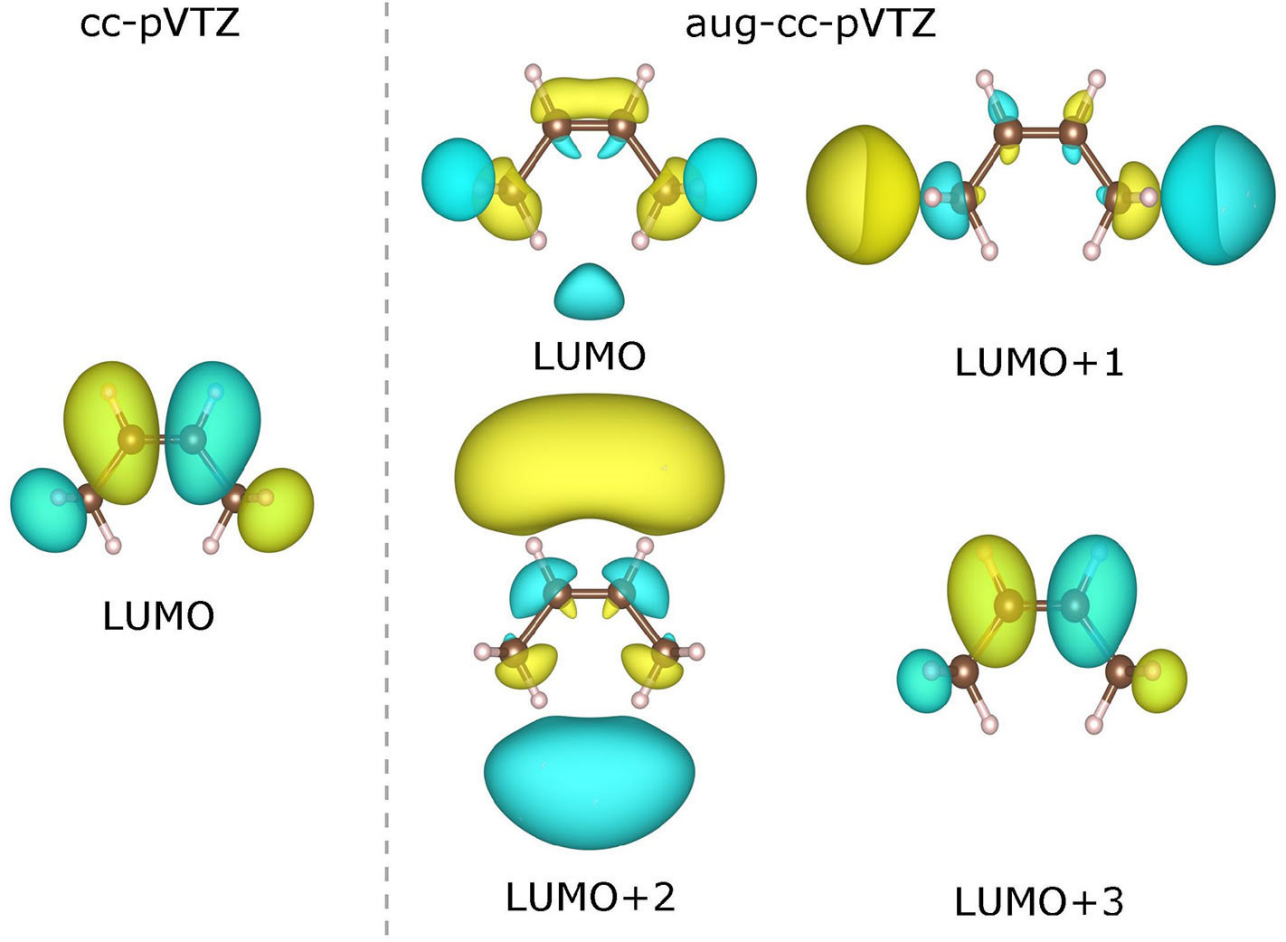
Contour plots of the lowest virtual Kohn–Sham (KS)/generalized KS (GKS) orbitals of the neutral *cis*-butene molecule calculated at the B3LYP/cc-pVTZ and B3LYP/aug-cc-pVTZ levels. The isosurfaces of ±0.022 a.u. are shaded in yellow and green, respectively).

As already been pointed out in Zhang et al. (2018), the use of a very diffuse basis set (such as aug-cc-pVTZ) may give rise to highly delocalized virtual KS/GKS orbitals with energies close to the molecular chemical potential. These orbitals are actually not relevant to the resonant state of the temporary anion of our interest, and should be left out of theoretical analysis. Therefore, we need to choose carefully the virtual orbital, which is genuinely pertinent to the formation of the temporary anion. For instance, in the case of a *cis*-butene molecule, the few lowest virtual orbitals calculated at the B3LYP/cc-pVTZ and B3LYP/aug-cc-pVTZ levels are depicted in Figure 6. Apparently, with the B3LYP/aug-cc-pVTZ method, the three lowest virtual orbitals (from LUMO to LUMO+2) are rather diffuse. Occupation on any of these orbitals by an excess electron will lead to an unbound state. Therefore, these orbitals are not relevant to the formation of the temporary anion. By scrutinizing the spatial distribution of the virtual KS/GKS orbitals, it is recognized that ϕ_LUMO+3_(**r**) would give rise to the resonant state of the temporary anion, as it exhibits a same shape as ϕ_LUMO_(**r**) obtained with the cc-pVTZ basis set. In such a case, instead of using Equation (16), the EA is predicted by $A=-\varepsilon_{\text{LUMO}+3}^{\text{GSC}-\text{B}3\text{LYP}}=-\left(\varepsilon_{\text{LUMO}+3}^{\text{B}3\text{LYP}}+\Delta \varepsilon_{\text{LUMO}+3}^{\text{GSC}}\right)$. Similarly, with the B3LYP/aug-cc-pVTZ method there are some other molecules for which a virtual orbital other than the LUMO should be chosen. The virtual orbital pertinent to the temporary anion is ϕ_LUMO+1_(**r**) for 9 molecules (aniline, propene, CO_2_, guanine, 1,4-cyclohexadiene, *cis*-1,2-difluoroethylene, 1,1-difluoroethylene, trifluoroethylene and tetrafluoroethylene), ϕ_LUMO+2_(**r**) for 3 molecules (trimethylethylene, propyne and acetylene), ϕ_LUMO+3_(**r**) for 3 molecules (pyrrole, *trans*-butene, and tetramethylethylene), and ϕ_LUMO+4_(**r**) for one molecule (cyclohexene).

As shown in Figure 5, unlike the QE-DFT method, increasing the size of basis set does not improve the accuracy of the ΔSCF method. This is because through the SCF calculation of the molecular anion by using a diffuse basis set, the excess electron is more inclined to reside on the delocalized orbital, which has a lower energy. Consequently, it is difficult to have the excess electron correctly occupying the virtual orbital that is pertinent to the resonant state of temporary anion. In contrast, the QE-DFT method in conjunction with the GSC approach does not require an SCF calculation for the anionic species, and is clearly more favorable for the prediction of resonance energies of temporary anions.

## 4. Conclusion

To summarize, we have calculated the QP, excitation, and resonance energies of molecules by employing the QE-DFT method. A non-empirical GSC approach is used to reduce the delocalization error associated with the DFAs by imposing an energy linearity condition for systems with a fractional number of electrons. The accuracy of the results obtained in this work with the GSC-DFAs is overall similar to that achieved in Mei et al. (2019) by the LOSC method (Li et al., 2017). For instance, the GSC-B3LYP yields an MAE of 0.36 eV and a mean sign error (MSE) of −0.16 eV for the 48 excitation energies of 16 molecules (see section 3.2). These errors are slightly smaller than the MAE of 0.49 eV and the MSE of −0.19 eV resulted by the LOSC-B3LYP method (Mei et al., 2019). The marginal superiority in the performance of the GSC is because of the explicit treatment of the relaxation of KS/GKS orbitals upon electron addition or removal. Relaxation of KS/GKS orbitals and electron density could also be included in the LOSC calculations (Su et al., 2020), which will further improve the accuracy of predicted QP energies. Moreover, as the size of the molecule increases to a certain extent, the energies at integer electron numbers may become less accurate. For such systems, the correction offered by the GSC approach may be inadequate, and the LOSC method with size-consistent corrections (Su et al., 2020; Yang et al., 2020) to DFA should be used.

For the various DFAs considered in this paper, the GSC-B3LYP yields the overall best performance. Our calculation results achieve at least the same level of accuracy as some more expensive methods, such as the *GW* method for QP energies, the TDDFT method for excitation energies, and the EOM-CC method for resonance energies. This thus affirms that it is entirely possible and practical to study excited-state properties within the framework of ground-state DFT. Despite the promising results, the prediction of singlet excitation energies still has plenty of room for improvement. This is because another source of error associated with the DFAs, the static correlation error, comes into play, which may be corrected by imposing a constancy condition on systems with fractional spins (Cohen et al., 2008b). Further work is needed along this direction.

## Data Availability Statement

All datasets generated for this study are included in the article/Supplementary Material.

## Author Contributions

XZ and WY conceived the project. XY conducted the numerical calculations. XY, XZ, and WY analyzed the data and WY wrote the paper. All authors contributed to the article and approved the submitted version.

## Conflict of Interest

The authors declare that the research was conducted in the absence of any commercial or financial relationships that could be construed as a potential conflict of interest.
