# Supplementary material for: Density Functional Prediction of Quasiparticle, Excitation, and Resonance Energies of Molecules With a Global Scaling Correction Approach
Source: Front Chem. 2020 Dec 8;8:588808. doi: 10.3389/fchem.2020.588808 (PMC7793789; doi:10.3389/fchem.2020.588808)
Supplement: Supplementary file 1 [file Data_Sheet_1.PDF]

Supplementary Material

Density functional prediction of quasiparticle, excitation and resonance energies of molecules with a global scaling correction approach

Xiaolong Yang [1], **Xiao Zheng** [1,*] **and Weitao Yang** [2,3,*]

[1]*Hefei National Laboratory for Physical Sciences at the Microscale and Synergetic Innovation Center of Quantum Information and Quantum Physics, University of Science and Technology of China, Hefei, Anhui 230026, China*
[2]*Department of Chemistry, Duke University, Durham, NC 27708, USA*
[3]*Key Laboratory of Theoretical Chemistry of Environment, School of Chemistry and Environment, South China Normal University, Guangzhou, China*

*__Correspondence__:
Xiao Zheng
xz58@ustc.edu.cn
Weitao Yang
weitao.yang@duke.edu

1 QP ENERGIES

1.1 Quasihole energies of molecules

Table S1 shows the occupied KS orbital energies from the calculation of density functional approximations (DFAs) and GSC-DFAs methods. 56 occupied orbital of 12 molecules are test in this work. We adopt GSC-DFAs up to second-order change in density for calculation. The basis set used for calculation is aug-cc-pVTZ.

Table S1: Mean absolute errors (MAEs) between the calculated and experimental QP energies of molecules. QP energies is calculated by using DFAs and GSC-DFAs methods. All energies are in units of eV. The experimental data were obtained from (Schmidt, 1977) and (Chong et al., 2002) and reference therein.

Molecule	MO	Exp	LDA	BLYP	B3LYP	PBE	GSC-LDA	GSC-BLYP	GSC-B3LYP	GSC-PBE
Cyanogen	$1\Pi_g$	13.51	9.59	9.18	10.38	9.35	13.41	12.98	12.96	13.17
	$5\Sigma_g$	14.49	10.17	9.99	11.70	10.07	14.05	13.86	14.45	13.97
	$4\Sigma_u$	14.86	10.44	10.28	12.02	10.39	14.31	14.14	14.80	14.27
	$1\Pi_u$	15.60	11.60	11.18	12.59	11.35	15.40	15.02	15.41	15.21
	$4\Sigma_g$*	22.80	18.95	18.73	20.69	18.85	24.00	23.76	21.07	23.89
	MAE		4.10	4.38	2.78	4.25	0.49	0.68	0.42	0.59
CO	5Σ	14.01	9.11	9.00	10.52	9.33	14.27	14.15	14.19	14.20
	1Π	16.91	12.13	11.72	13.20	11.87	17.74	17.31	16.87	17.48
	$4\Sigma_g$*	19.72	14.20	14.00	15.91	14.14	20.14	19.90	19.93	20.04
	MAE		5.07	5.31	3.67	5.10	0.50	0.24	0.14	0.36
Acetylene	$1\Pi_u$	11.49	7.38	7.05	8.20	7.20	11.75	11.39	11.30	11.58
	$3\Sigma_g$	16.70	12.24	12.16	13.74	12.20	16.74	16.66	17.00	16.71
	$2\Sigma_u$	18.70	13.99	13.93	15.62	14.04	18.40	18.41	19.00	18.54
	$2\Sigma_g$*	23.50	18.53	18.45	20.68	18.62	23.68	23.57	24.29	23.77
	MAE		4.56	4.70	3.04	4.58	0.18	0.13	0.40	0.13
Water	$1B_1$	12.62	7.37	7.19	8.80	7.22	13.21	13.00	12.55	13.08
	$3A_1$	14.74	9.28	9.14	10.75	9.20	15.15	14.99	14.57	15.08
	$1B_2$	18.55	13.34	13.21	14.78	13.27	19.27	19.07	18.89	19.17
	MAE		5.31	5.46	3.86	5.41	0.57	0.38	0.19	0.47
Ethylene	$1B_{3u}$	10.68	6.96	6.61	7.67	6.78	11.02	10.65	10.54	10.85
	$1B_{3g}$	12.80	8.47	8.47	9.80	8.50	12.37	12.36	12.71	12.41
	$3A_g$	14.80	10.30	10.16	11.64	10.22	14.54	14.40	14.62	14.47
	$1B_{2u}$	16.00	11.53	11.37	12.90	11.47	15.55	15.38	15.86	15.51
	$2B_{1u}$	19.10	14.18	14.12	15.91	14.25	18.28	18.21	18.96	18.36
	$2A_g$*	23.60	18.74	18.63	20.85	18.82	23.34	23.22	24.11	23.43
	MAE		4.47	4.60	3.04	4.49	0.43	0.46	0.20	0.38
Ammonia	$3A_1$	10.80	6.14	5.96	7.35	6.03	11.22	11.02	10.65	11.13
	$1E$	16.00	11.42	11.32	12.79	11.39	16.68	16.57	16.50	16.66
	MAE		4.62	4.78	3.33	4.69	0.55	0.40	0.32	0.50
Acetonitrile	$2E$	12.46	8.30	7.99	9.24	8.13	12.53	12.24	12.17	12.39
	$7A_1$	13.17	8.58	8.45	10.09	8.52	13.54	13.40	13.19	13.48
	$1E$*	15.70	11.59	11.39	12.86	11.50	15.83	15.68	15.92	15.79

Table.S1. (Continued.)

Molecule	MO	Exp	LDA	BLYP	B3LYP	PBE	GSC-LDA	GSC-BLYP	GSC-B3LYP	GSC-PBE
	$6A_1$*	17.40	12.89	12.71	14.29	12.83	17.19	16.99	17.27	17.11
	$5A_1$*	24.90	19.36	19.21	21.36	19.39	23.80	23.65	24.52	23.85
	MAE		4.58	4.78	3.16	4.65	0.38	0.43	0.21	0.36
Fluoromethane	$2E$	13.10	8.17	8.03	9.67	8.06	13.00	12.92	12.94	12.96
	$1E$*	17.00	11.76	11.56	13.27	11.63	16.32	16.10	16.61	16.19
	$5A_1$	17.00	12.14	11.83	13.42	11.94	17.22	16.93	16.74	17.06
	$4A_1$*	23.40	17.25	17.15	19.24	17.30	21.95	21.87	22.71	22.04
	MAE		5.30	5.49	3.73	5.39	0.62	0.67	0.37	0.59
Benzene	E_{1g}	9.24	6.54	6.14	7.08	6.33	9.58	9.17	9.17	9.39
	A_{2u}	12.25	8.28	8.16	9.51	8.22	11.31	11.19	11.74	11.25
	MAE		3.34	3.60	2.45	3.47	0.64	0.57	0.29	0.58
Naphthalene	A_u	8.15	5.71	5.30	6.14	5.50	8.18	7.77	7.85	7.97
	B_{1u}	8.87	6.43	6.01	6.89	6.22	8.98	8.55	8.59	8.77
	B_{2g}	10.08	7.36	6.94	8.00	7.15	9.91	9.48	9.79	9.70
	B_{3g}	10.83	8.01	7.85	9.15	7.91	10.55	10.40	10.97	10.47
	MAE		2.61	2.96	2.26	2.79	0.15	0.43	0.25	0.26
Furan	$1A_2$	9.00	5.89	5.53	6.51	5.69	9.24	8.86	8.83	9.04
	$2B_1$	10.40	7.08	6.71	7.85	6.88	10.56	10.17	10.15	10.36
	$9A_1$	13.00	9.08	8.82	10.35	8.94	12.93	12.68	12.88	12.80
	$8A_1$*	13.80	9.77	9.56	11.00	9.65	13.33	13.12	13.49	13.21
	$6B_2$	14.40	10.02	9.90	11.36	9.94	13.50	13.37	13.88	13.42
	$5B_2$*	15.25	10.97	10.79	12.26	10.89	14.41	14.24	14.76	14.34
	$1B_1$*	15.60	11.26	10.84	12.43	11.03	15.12	14.69	14.93	14.90
	$7A_1$*	17.50	13.44	13.17	14.89	13.33	16.85	16.54	17.39	16.72
	$6A_1$*	18.80	14.00	13.85	15.63	13.99	17.52	17.35	18.23	17.50
	$4B_2$*	19.70	14.92	14.64	16.44	14.82	18.49	18.19	19.02	18.38
	$3B_2$*	23.00	18.51	18.28	20.41	18.49	22.20	21.93	23.03	22.17
	MAE		4.14	4.40	2.85	4.25	0.65	0.85	0.36	0.69
HCOOH	$10A'$	11.50	7.13	6.89	8.38	6.94	11.74	11.50	11.22	11.58
	$2A''$	12.60	8.45	8.13	9.52	8.23	12.87	12.52	12.26	12.64
	$9A'$*	14.80	10.06	9.88	11.50	9.94	14.35	14.15	14.53	14.23
	$1A''$*	15.80	11.50	11.11	12.68	11.24	15.81	15.40	15.81	15.56
	$8A'$*	17.10	12.12	11.90	13.74	12.03	16.65	16.40	16.91	16.55
	$7A'$*	17.80	13.14	12.91	14.59	12.99	17.59	17.35	17.81	17.45
	$6A'$*	22.00	16.88	16.67	18.54	16.80	21.63	21.38	21.90	21.53
	MAE		4.62	4.87	3.24	4.77	0.29	0.41	0.17	0.33
Total MAE			4.37	4.60	3.05	4.47	0.45	0.52	0.28	0.46

1.2 Photoemission spectrum of molecules not shown in the main text

Figure S1 - S12 show the photoemission spectrum (PES) of 12 test molecules. Maleic anhydride and benzoquinone have been shown in main text. The test molecules were from the test set proposed by Marom (Knight et al., 2016). Experimental PES and electron affinity (EA) were reproduced from literature as reference. Experimental EA was broadened with Gaussian expansion with 0.2 eV to plot a peak in the experimental spectrum. To obtain PES from the orbital energies of GW method and DFT, the same type of Gaussian function were used. QP energies from scGW and G_0W_0@PBE were also obtained from (Knight et al., 2016) and used to plot PES for comparison. For orbital energies from DFT, the basis set of cc-pVTZ were used.

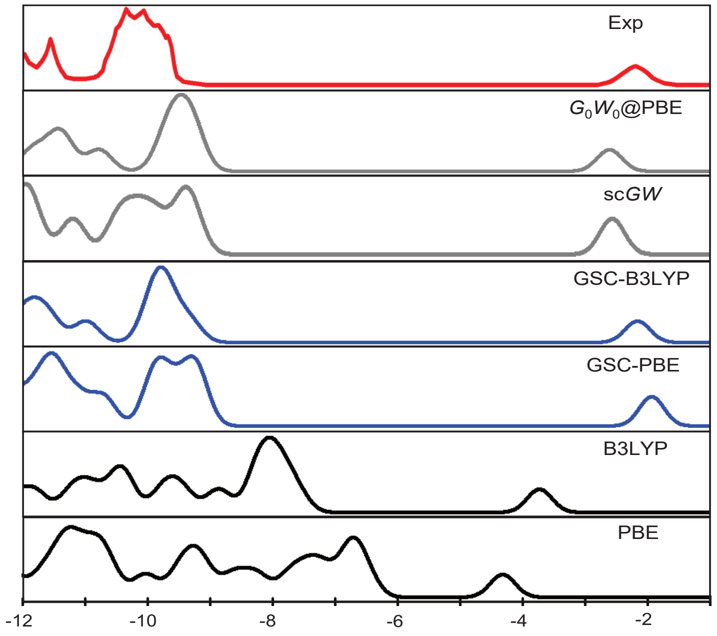

Figure S1: Photoemission spectrum of dichlone. Experimental spectrum was reproduced from (Kimura, 1981) and additional experimental EA value is taken from (Heinis et al., 1988)

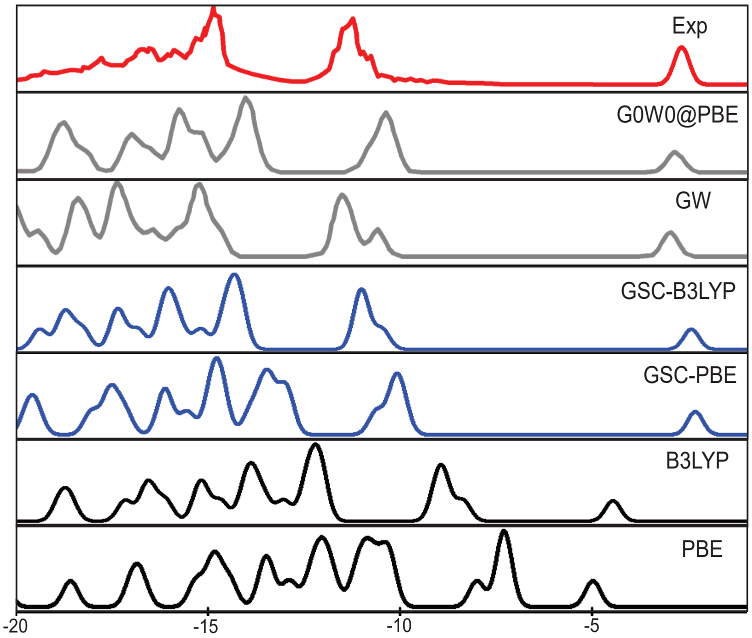

Figure S2: Photoemission spectrum of F_4-benzoquinone. Experimental spectrum was obtained from (Brundle et al., 1972) and additional experimental EA value is taken from (Heinis et al., 1988)

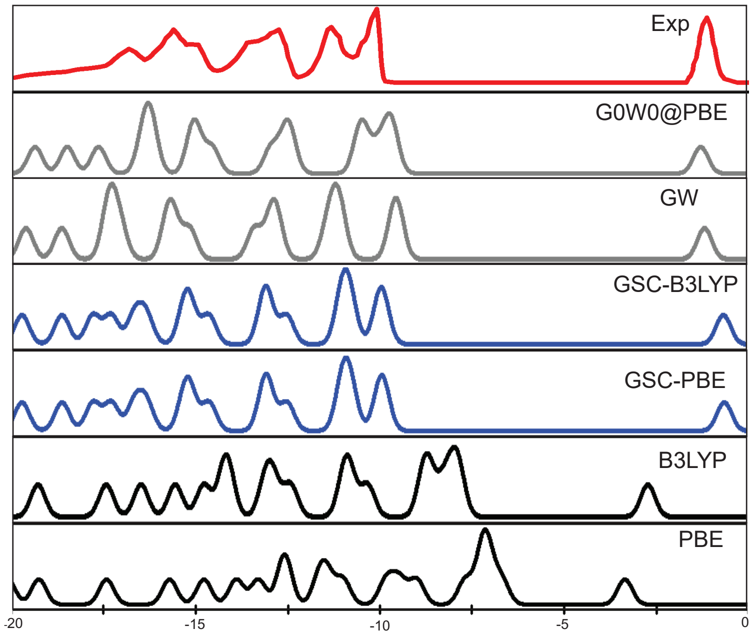

Figure S3: Photoemission spectrum of nitrobenzene. Experimental spectrum was obtained from (Rabalais, 1972) and additional experimental EA value is taken from (Desfrancois et al., 1999)

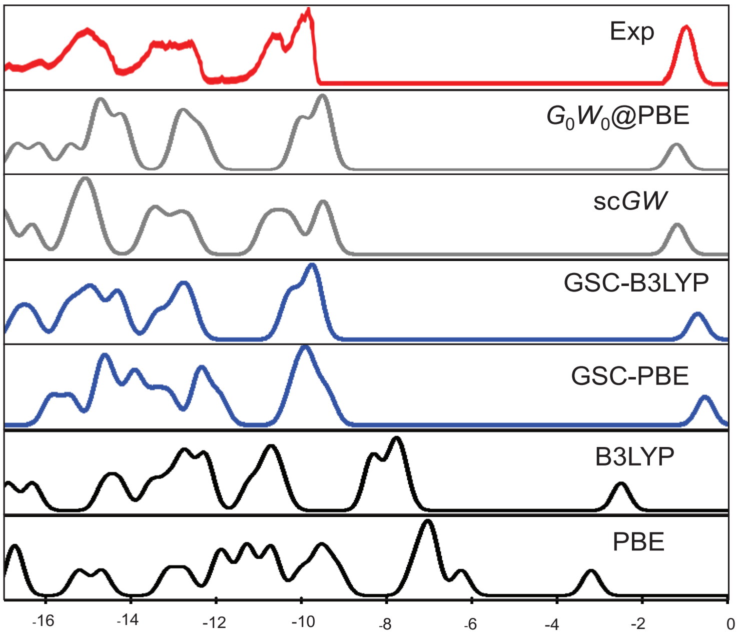

Figure S4: Photoemission spectrum of phthalimide. Experimental spectrum was obtained from (Galasso et al., 1977) and additional experimental EA value is taken from (Paul and Kebarle, 1989)

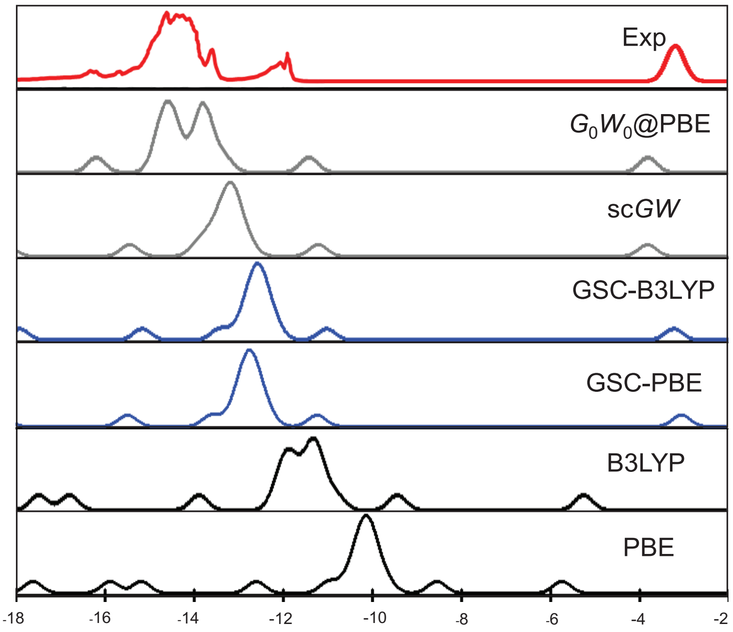

Figure S5: Photoemission spectrum of TCNE. Experimental spectrum was obtained from (Ikemoto et al., 1974) and additional experimental EA value is taken from (Khuseynov et al., 2012)

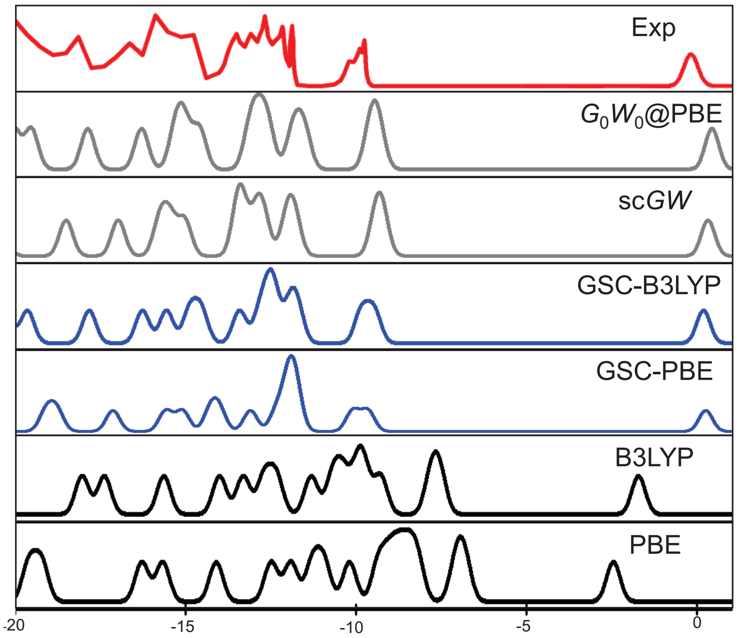

Figure S6: Photoemission spectrum of benzonitrile. Experimental spectrum was obtained from (Kimura, 1981) and additional experimental EA value is taken from (Zlatkis et al., 1983)

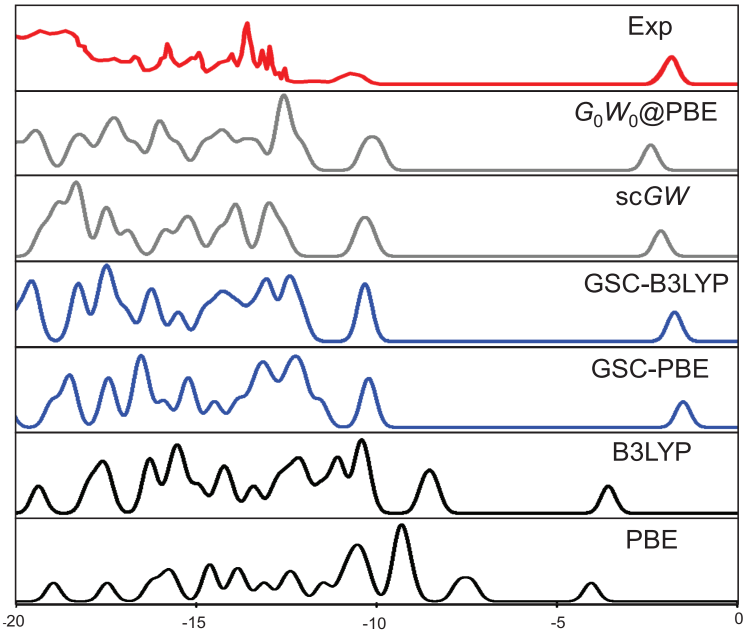

Figure S7: Photoemission spectrum of F_4-benzenedicarbonitrile. Experimental spectrum was obtained from (Neijzen and De Lange, 1978) and additional experimental EA value is taken from (Chowdhury et al., 1986)

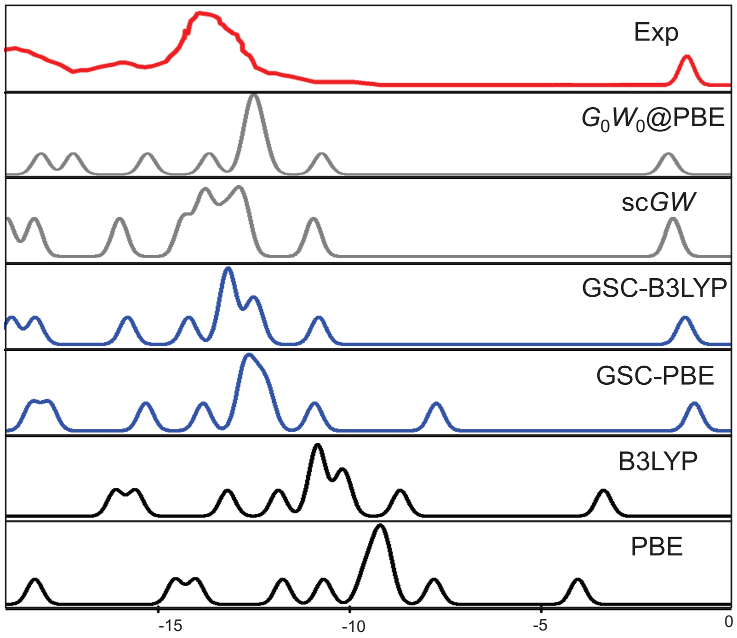

Figure S8: Photoemission spectrum of fumaronitrile. Experimental spectrum was obtained from (Fujikawa et al., 1976) and additional experimental EA value is taken from (Chowdhury and Kebarle, 1986)

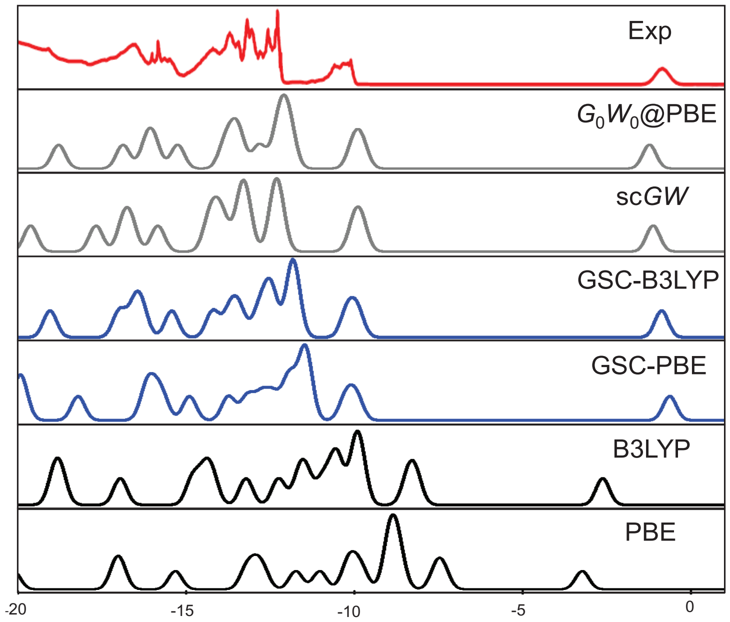

Figure S9: Photoemission spectrum of mDCNB. Experimental spectrum was obtained from (Neijzen and De Lange, 1978) and additional experimental EA value is taken from (Chowdhury and Kebarle, 1986)

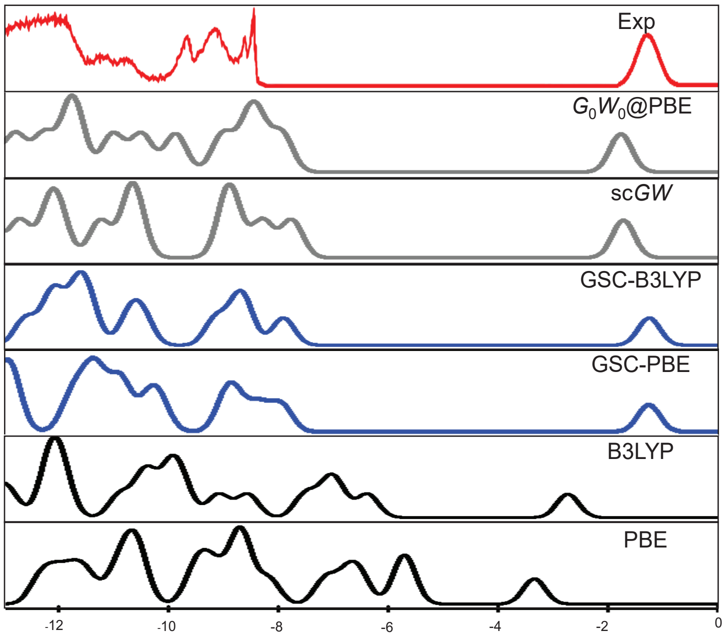

Figure S10: Photoemission spectrum of phenazine. Experimental spectrum was obtained from (Maier et al., 1975) and additional experimental EA value is taken from (Dillow and Kebarle, 1989)

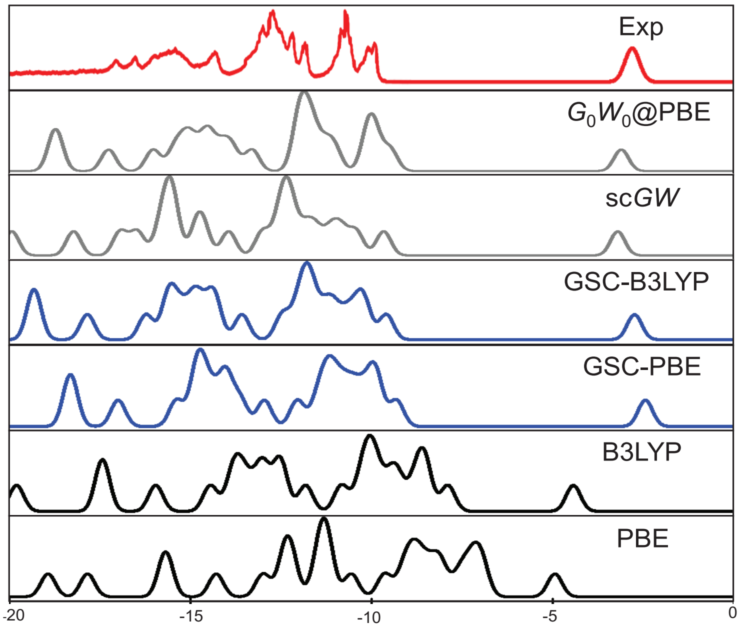

Figure S11: Photoemission spectrum of Cl_4-benzoquinone. Experimental spectrum was obtained from (Dougherty and McGlynn, 1977) and additional experimental EA value is taken from (Heinis et al., 1988)

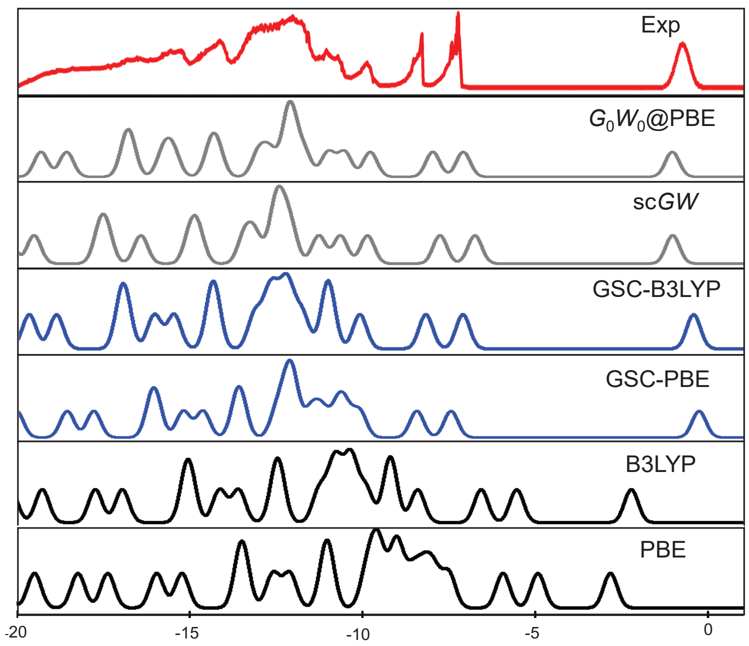

Figure S12: Photoemission spectrum of azulene. Experimental spectrum was obtained from (Dougherty et al., 1980) and additional experimental EA value is taken from (Ando et al., 2008)

2 ENERGIES OF LOW-LYING EXCITED STATES

Table S2-S3 show the detailed 48 low-lying excitation energies from different methods on 16 molecular test set. Geometries were obtained from (Yang et al., 2014). The basis set used for calculation was 6-311++G(3df, 3pd).The orbital relaxation is considered up to second-order for GSC-B3LYP, and first-order for other GSC-DFAs, respectively.

Table S2: Low-lying vertical excitation energies obtained from (N-1)-electron system with DFAs, GSC-DFAs and TDDFT method. Reference data were obtained from (Schreiber et al., 2008) and the data of TDDFT-B3LYP are taken from (Mei et al., 2018). All energies are in units of eV.

Molecule	MO	Ref	LDA	BLYP	B3LYP	PBE	GSC-LDA	GSC-BLYP	GSC-B3LYP	GSC-PBE	TDDFT-B3LYP
Ethene	$^3B_{1u}$	4.50	4.90	4.61	4.43	4.41	4.91	4.64	4.43	4.45	4.05
Ethene	$^1B_{1u}$	7.80	7.27	7.46	7.75	7.69	6.67	6.76	7.09	7.01	7.38
Furan	$^3B_2$	4.17	4.31	4.05	3.95	4.00	4.28	4.04	3.94	3.99	3.70
Furan	$^3A_2$	5.99*	5.99	5.78	5.84	5.74	5.91	5.70	5.64	5.75	5.48
Furan	$^1B_2$	6.32	5.60	5.73	6.11	5.85	5.39	5.47	5.87	5.60	5.94
Furan	$^1A_2$	6.03*	6.91	6.91	7.27	6.98	6.67	6.67	7.11	6.71	5.51
Benzoquinone	$^3B_{1g}$	2.51	1.60	1.68	2.08	1.62	1.57	1.64	2.10	1.57	1.93
Benzoquinone	$^3B_{3u}$	5.38*	4.66	4.76	5.40	4.70	5.28	4.64	5.42	4.57	5.18
Benzoquinone	$^1B_{1g}$	2.78	1.94	1.95	2.41	1.88	1.89	1.90	2.36	1.83	2.43
Benzoquinone	$^1B_{3u}$	5.60	4.78	4.85	5.49	4.80	5.40	4.72	5.52	4.67	5.38
cyclopentadiene	$^3B_2$	3.25	3.46	3.21	3.12	3.16	3.40	3.15	3.09	3.09	2.74
cyclopentadiene	$^3A_2$	5.61*	5.85	5.65	5.76	5.62	5.76	5.55	5.67	5.53	5.09
cyclopentadiene	$^1B_2$	5.55	4.66	4.74	5.06	4.84	4.44	4.51	4.92	4.63	4.95
cyclopentadiene	$^1A_2$	5.65*	6.77	6.79	7.14	6.86	6.52	6.48	6.85	6.56	5.11
butadiene	^3B_u	3.20	3.40	3.22	3.19	3.13	3.39	3.21	3.25	3.13	2.79
butadiene	^3B_g	6.22*	6.05	5.86	6.02	5.84	5.91	5.70	5.94	5.70	5.67
butadiene	^1B_u	6.18	4.72	4.86	5.39	4.98	4.49	4.60	5.34	4.73	5.56
butadiene	^1B_g	6.26*	6.86	6.80	7.04	6.90	6.51	6.36	6.67	6.48	5.70
hexatriene	^3B_u	2.40	2.62	2.49	2.51	2.45	2.63	2.49	2.60	2.45	2.12
hexatriene	^3A_u	5.68*	4.95	4.82	5.10	4.81	4.88	4.74	5.19	4.74	5.22
hexatriene	^1B_u	5.10	3.54	3.66	4.22	3.74	3.45	3.53	4.38	3.61	4.60
hexatriene	^1A_u	5.71*	5.60	5.58	6.05	5.67	5.42	5.36	6.00	5.44	5.24
octetraene	^3B_u	2.20	2.16	2.05	2.11	2.02	2.06	2.06	2.20	2.03	1.71
octetraene	^1B_u	4.66	2.86	2.96	3.52	3.00	2.80	2.89	3.80	2.93	3.96
cyclopropene	$^3B_2$	4.34	4.38	4.22	4.19	4.13	4.36	4.21	4.11	4.12	3.70
cyclopropene	$^1B_2$	7.06	5.97	6.29	6.69	6.41	5.59	5.83	6.32	5.98	6.09
norbornadiene	$^3A_2$	3.72	3.73	3.62	3.71	3.59	3.62	3.50	3.59	3.48	3.10
norbornadiene	$^1A_2$	5.34	4.56	4.64	4.90	4.17	4.36	4.42	4.65	4.48	4.70
s-tetrazine	^3B_u	1.89	1.21	1.32	1.63	1.27	1.24	1.33	1.54	1.28	1.47
s-tetrazine	^3A_u	3.52	2.58	2.98	3.45	2.93	2.75	2.88	3.30	2.82	3.15
s-tetrazine	^1B_u	2.24	1.74	1.77	2.17	1.72	1.72	1.75	2.06	1.70	2.27
s-tetrazine	^1A_u	3.48	3.06	3.13	3.59	3.07	2.97	3.02	3.45	2.96	3.54
formaldehyde	$^3A_2$	3.50	3.38	3.37	3.17	3.32	3.33	3.29	3.46	3.25	3.10

Table S2. (Continued.)

Molecule	MO	Ref	LDA	BLYP	B3LYP	PBE	GSC-LDA	GSC-BLYP	GSC-B3LYP	GSC-PBE	TDDFT-B3LYP
formaldehyde	$^3A_2$	3.88	3.89	3.63	3.41	3.58	3.79	3.53	3.72	3.51	3.83
acetone	$^3B_1$	4.05	4.09	3.95	3.78	3.93	4.02	3.88	4.08	3.85	3.68
acetone	$^3B_2$	5.87^*	8.38	7.95	7.47	8.08	6.71	6.18	6.36	6.31	5.71
acetone	$^1B_1$	4.40	4.50	4.19	4.01	4.16	4.42	4.10	4.33	4.09	4.30
acetone	$^1B_2$	5.92^*	8.46	8.02	7.54	8.17	6.77	6.24	6.39	6.39	5.77
pyridine	$^3B_1$	4.25	4.43	4.37	4.42	4.38	N/A	4.25	N/A	4.12	4.05
pyridine	$^3A_2$	5.28	5.15	4.99	4.60	5.17	N/A	4.83	N/A	4.97	4.96
pyridine	$^1B_1$	4.59	4.89	4.80	5.54	4.74	N/A	4.65	N/A	4.58	4.76
pyridine	$^1A_2$	5.11	5.32	5.36	6.40	5.21	N/A	5.17	N/A	5.01	5.10
pyridazine	$^1B_1$	3.78	3.64	3.61	3.64	4.08	3.44	3.41	3.46	3.85	3.60
pyridazine	$^1A_2$	4.32	4.26	4.25	4.25	4.21	4.03	4.01	4.11	3.97	4.19
pyrazine	^1B_u	3.95	3.52	3.53	3.90	3.49	3.44	3.45	3.77	5.40	3.93
pyrazine	^1A_u	4.81	4.34	4.39	4.76	4.33	4.80	4.25	4.63	4.19	4.69
pyrimidine	$^1B_1$	4.55	4.01	3.97	4.17	4.20	4.02	3.85	4.15	4.08	4.25
pyrimidine	$^1A_2$	4.91	4.40	4.37	4.59	4.32	4.26	4.23	4.56	4.19	4.60
MAE			0.58	0.53	0.42	0.54	0.59	0.53	0.36	0.53	0.38

Table S3: MAE and mean sign errors (MSEs) of 48 low-lying excitation energies obtained from $(N-1)$-electron system with DFAs, GSC-DFAs and TDDFT (B3LYP). T1 stands for triplet HOMO to LUMO excitation, and T2 stands for triplet HOMO to LUMO+1 excitation. The analogous notation is used for S1 and S2, which stand for singlet excitations. All energies are in units of eV.

	T1		T2		S1		S2		Total	
method	MAE	MSE	MAE	MSE	MAE	MSE	MAE	MSE	MAE	MSE
LDA	0.24	-0.02	0.65	0.04	0.73	-0.68	0.70	-0.27	0.58	-0.16
BLYP	0.19	-0.14	0.63	-0.10	0.68	-0.59	0.65	0.81	0.53	-0.83
PBE	0.23	-0.2	0.64	-0.08	0.63	-0.57	0.69	0.25	0.53	-0.20
B3LYP	0.17	-0.13	0.43	0.01	0.45	-0.33	0.67	-0.58	0.42	-0.01
GSC-LDA	0.27	-0.06	0.54	-0.26	0.92	-0.91	0.53	-0.01	0.59	-0.20
GSC-BLYP	0.21	-0.18	0.49	-0.42	0.85	-0.85	0.47	-0.12	0.53	-0.43
GSC-PBE	0.25	-0.24	0.52	-0.41	0.77	-0.76	0.53	-0.11	0.53	-0.41
GSC-B3LYP	0.18	-0.14	0.28	-0.11	0.49	-0.49	0.43	0.26	0.36	-0.16
TDDFT-B3LYP	0.45	-0.45	0.39	-0.39	0.38	-0.35	0.28	0.27	0.38	-0.37

3 RESONANCE ENERGIES

Table S4-S6 show the detailed the negative electron affinities (EAs) for 64 molecules from DFAs, GSC-DFAs, and ΔSCF methods. The basis set used for the calculation is cc-pVTZ in Table S4 and aug-cc-pVTZ in Table S5. The test set of 38 molecules are come from our earlier study (Zhang et al., 2018). Geometries of other molecules in the extended test set were also optimized from B3LYP/6-311+G** level with the Gaussian 09 suite of programs(Frisch et al., 2009).

Table S4: Negative EAs obtained from DFAs, GSC-DFAs and ΔSCF methods (PBE and B3LYP) compared with the experimental reference. The basis set cc-pVTZ is used. All energies are in units of eV. The experimental values of the test set are taken from (Zhang et al., 2018; Tozer and De Proft, 2005; Chiu et al., 1979; Jordan and Burrow, 1978; Ng et al., 1983) and reference therein.

Molecule	Exp	LDA	BLYP	B3LYP	PBE	GSC-LDA	GSC-BLYP	GSC-B3LYP	GSC-PBE	ΔSCF-PBE	ΔSCF-B3LYP
1,1-Dichloroethylene	-0.75	1.81	1.44	0.83	1.58	-1.14	-1.51	-1.47	-1.39	-1.52	-1.44
1,3-Cyclohexadiene	-0.80	1.87	1.49	0.90	1.64	-0.80	-1.17	-1.15	-1.03	-1.13	-1.17
Acetaldehyde	-1.19	2.13	1.76	0.94	1.87	-1.45	-1.81	-1.92	-1.72	-1.84	-1.78
Adenine	-0.64	1.76	1.34	0.80	1.48	-0.71	-1.12	-1.03	-0.99	-1.08	-1.13
Bromobenzene	-0.70	1.77	1.36	0.79	1.52	-0.78	-1.19	-1.17	-1.04	-1.12	-1.19
Chlorobenzen	-0.75	1.75	1.34	0.77	1.50	-0.85	-1.26	-1.24	-1.11	-1.23	-1.25
Chloroethylene	-1.29	1.52	1.15	0.50	1.29	-1.64	-2.00	-1.99	-1.89	-1.98	-1.93
Chloromethane	-1.30	0.72	0.50	-0.17	0.55	-2.40	-2.44	-2.30	-2.48	-2.26	-2.28
cis-Dichloroethylene	-1.12	1.73	1.35	0.75	1.50	-1.25	-1.63	-1.58	-1.50	-1.63	-1.55
Cytosine	-0.36	2.09	1.69	1.16	1.82	-0.61	-1.01	-0.89	-0.89	-1.03	-0.94
Ethylene	-1.78	1.15	0.79	0.09	0.91	-2.27	-2.60	-2.58	-2.51	-2.52	-2.51
Fluorobenzene	-0.87	1.68	1.27	0.72	1.42	-1.04	-1.44	-1.37	-1.30	-1.39	-1.39
Naphthalene	-0.20	2.26	1.83	1.34	2.02	-0.04	-0.46	-0.38	-0.29	-0.37	-0.46
Norbornadiene	-1.04	1.53	1.12	0.49	1.27	-1.05	-1.44	-1.52	-1.31	-1.39	-1.52
Pyrazine	-0.07	2.77	2.35	1.75	2.51	-0.17	-0.58	-0.48	-0.44	-0.56	-0.52
Pyridazine	-0.32	2.75	2.33	1.73	2.48	-0.18	-0.59	-0.49	-0.46	-0.56	-0.53
Pyrimidine	-0.25	2.54	2.11	1.50	2.27	-0.39	-0.81	-0.75	-0.67	-0.76	-0.76
Styrene	-0.25	2.21	1.79	1.27	1.96	-0.18	-0.59	-0.53	-0.43	-0.52	-0.60
Thiophene	-1.17	1.51	1.15	0.61	1.30	-1.29	-1.64	-1.53	-1.51	-1.59	-1.56
$trans$-Dichloroethylene	-0.82	1.86	1.48	0.88	1.62	-1.10	-1.47	-1.44	-1.35	-1.49	-1.41
Trichloroethylene	-0.58	2.02	1.64	1.07	1.78	-0.79	-1.17	-1.10	-1.03	-1.19	-1.08
Uracil	-0.21	2.52	2.10	1.56	2.23	-0.25	-0.66	-0.57	-0.54	-0.68	-0.61
1,2,4-Trimethylbenzene	-1.07	1.12	0.73	0.17	0.88	-1.25	-1.63	-1.68	-1.49	-1.62	-1.69
Acetone	-1.51	1.75	1.40	0.61	1.52	-1.49	-1.82	-1.95	-1.72	-1.80	-1.81
Aniline	-1.13	1.18	0.78	0.23	0.93	-1.43	-1.82	-1.78	-1.69	-1.77	-1.80
Anisole	-1.09	1.32	0.92	0.37	1.07	-1.24	-1.64	-1.62	-1.50	-1.61	-1.64
cis-Butene	-2.22	0.47	1.34	-0.55	0.26	-2.46	-2.79	-2.87	-2.67	-2.44	-2.78
Cyclohexene	-2.07	0.57	0.24	-0.40	0.36	-2.09	-2.38	-2.35	-2.31	-2.07	-2.20
Furan	-1.76	0.94	0.58	-0.03	0.70	-2.07	-2.41	-2.31	-2.31	-2.41	-2.35
m-Xylene	-1.06	1.23	0.85	0.29	1.00	-1.21	-1.59	-1.61	-1.45	-1.55	-1.61
o-Xylene	-1.12	1.14	0.76	0.19	0.92	-1.32	-1.70	-1.70	-1.55	-1.66	-1.72
Phenol	-1.01	1.40	1.00	0.45	1.15	-1.27	-1.67	-1.61	-1.53	-1.62	-1.63

Table.S4. (Continued.)

Molecule	Exp	LDA	BLYP	B3LYP	PBE	GSC-LDA	GSC-BLYP	GSC-B3LYP	GSC-PBE	ΔSCF-PBE	ΔSCF-B3LYP
Propene	-1.99	0.81	0.46	-0.23	0.59	-2.33	-2.66	-2.71	-2.56	-2.54	-2.63
Pyrrole	-2.38	0.15	0.20	-0.79	-0.08	-2.77	-3.11	-2.24	-3.01	-2.29	-2.40
trans-Butene	-2.10	0.52	0.18	-0.51	0.31	-2.40	-2.73	-2.83	-2.62	-2.66	-2.74
Trimethylethylene	-2.24	0.53	0.22	-0.43	0.35	-2.12	-2.41	-2.50	-2.31	-2.24	-2.42
CO_2	-3.80	0.48	0.10	-0.70	0.15	-3.80	-4.14	-4.12	-4.12	-3.48	-3.45
Guanine	-0.46	1.42	1.02	0.47	1.14	-1.03	-1.38	-1.32	-1.30	-1.25	-1.34
Monofluoroethylene	-1.91	1.08	0.73	0.05	0.83	-2.38	-2.71	-2.66	-2.64	-2.67	-2.59
trans-1,2-difluoroethylene	-1.84	1.10	0.75	0.10	0.83	-2.39	-2.73	-2.67	-2.67	-2.75	-2.59
cis-1,2-difluoroethylene	-2.18	0.97	0.61	0.04	0.69	-2.54	-2.88	-2.82	-2.82	-2.81	-2.73
1,1-Difluoroethylene	-2.39	0.32	0.52	-0.15	0.60	-2.62	-2.98	-2.90	-2.92	-3.00	-2.84
Trifluoroethylene	-2.45	0.86	0.49	-0.15	0.54	-2.70	-3.06	-2.99	-3.02	N/A	-2.91
Tetrafluoroethylene	-3.00	0.67	0.29	-0.35	0.32	-2.95	-3.33	-3.29	-3.31	-3.49	-3.19
Nitrogen	-2.20	2.06	1.72	0.80	1.78	-2.58	-2.89	-2.90	-2.86	-2.99	-2.80
Formaldehyde	-0.86	2.76	2.38	1.53	2.49	-1.26	-1.61	-1.61	-1.54	-1.55	-1.47
Butadiene	-0.62	2.05	1.66	1.06	1.81	-0.79	-1.17	-1.09	-1.03	-1.10	-1.13
Biphenyl	-0.30	1.98	1.55	1.05	1.74	-0.11	-0.54	-0.53	-0.37	-0.46	-0.60
Trichloromethane	-0.35	2.36	2.08	1.40	2.19	-0.48	-0.73	-0.75	-0.65	-0.75	-0.78
Dichlorofluoromethane	-0.96	1.84	1.55	0.86	1.64	-1.23	-1.48	-1.46	-1.42	-1.45	-1.44
Dichlorodifluoromethane	-0.98	2.22	1.92	1.23	2.00	-0.92	-1.20	-1.19	-1.14	-1.30	-1.22
Dichloromethane	-1.23	1.59	1.33	0.64	1.41	-1.41	-1.61	-1.59	-1.56	-1.55	-1.58
Benzene	-1.15	1.36	0.96	0.38	1.12	-1.36	-1.75	-1.70	-1.61	-1.69	-1.73
CO	-1.80	2.12	1.79	0.95	1.86	-2.22	-2.51	-2.48	-2.48	-2.44	-2.33
Cyanogen	-0.58	3.83	3.41	2.78	3.55	0.35	0.05	0.10	0.07	-0.03	0.07
Propyne	-2.95	-0.11	-0.41	-1.00	0.31	-3.17	-3.42	-2.17	-3.36	-2.62	-2.69
Butadiyne	-1.00	2.02	1.63	1.05	1.78	-0.97	-1.35	-1.24	-1.22	-1.27	-1.27
Tetramethylethylene	-2.27	3.11	0.03	-0.62	0.15	-2.17	-2.44	-2.54	-2.34	-2.14	-2.25
Acetylene	-2.60	0.37	0.03	0.67	0.13	-3.14	-3.44	-3.40	-3.38	-2.77	-2.93
Acrylonitrile	-0.21	2.94	2.53	1.92	2.68	-0.18	-0.56	-0.47	-0.44	-0.51	-0.48
1,4-Cyclohexadiene	-1.75	0.95	0.54	-0.15	0.69	-1.67	-2.08	-2.22	-1.94	-2.02	-2.19
Toluene	-1.11	1.31	0.91	0.34	1.07	-1.28	-1.67	-1.66	-1.53	-1.62	-1.67
Ethylbenzene	-1.17	1.31	0.90	0.33	1.14	-1.22	-1.62	-1.64	-1.31	-1.47	-1.64
Isopropylbenzene	-1.08	1.32	0.91	0.34	1.15	-1.14	-1.56	-1.59	-1.25	N/A	-1.60
Total MAE		2.85	2.49	1.81	2.57	0.23	0.54	0.52	0.43	0.47	0.48

Table S5: Negative EAs obtained from DFAs, GSC-DFAs and ΔSCF methods (PBE and B3LYP) compared with the experimental reference. The basis set aug-cc-pVTZ is used. All energies are in units of eV. The experimental values of the test set are taken from (Zhang et al., 2018; Tozer and De Proft, 2005; Chiu et al., 1979; Jordan and Burrow, 1978; Ng et al., 1983) and reference therein.

Molecule	Exp	LDA	BLYP	B3LYP	PBE	GSC-LDA	GSC-BLYP	GSC-B3LYP	GSC-PBE	ΔSCF-PBE	ΔSCF-B3LYP
1,1-Dichloroethylene	-0.75	1.93	1.60	1.02	1.72	-0.85	-1.12	-1.05	-1.04	-0.36	-0.44
1,3-Cyclohexadiene	-0.80	1.93	1.58	0.99	1.72	-0.65	-0.98	-0.96	-0.86	-0.34	-0.41
Acetaldehyde	-1.19	2.26	1.92	1.11	2.02	-1.18	-1.49	-1.58	-1.41	-0.61	-0.41
Adenine	-0.64	1.92	1.53	0.98	1.66	-0.45	-0.81	-0.75	-0.70	-0.09	-0.17
Bromobenzene	-0.70	1.84	1.45	0.90	1.61	-0.63	-1.00	-0.98	-0.86	-0.22	-0.31
Chlorobenzen	-0.75	1.82	1.43	0.88	1.59	-0.69	-1.06	-1.03	-0.92	-0.28	-0.35
Chloroethylene	-1.29	1.64	1.31	0.69	1.42	-1.35	-1.61	-1.54	-1.54	-0.44	-0.50
Chloromethane	-1.30	0.99	0.92	0.50	0.90	-1.42	-1.00	-0.60	-1.23	-0.37	-0.42
cis-Dichloroethylene	-1.12	1.81	1.45	0.85	1.59	-1.07	-1.39	-1.36	-1.28	-0.34	-0.40
Cytosine	-0.36	2.24	1.87	1.33	1.99	-0.36	-0.70	-0.62	-0.60	0.02	-0.03
Ethylene	-1.78	1.28	0.97	0.32	1.07	-1.90	-2.10	-1.99	-2.07	-0.51	-0.57
Fluorobenzene	-0.87	1.75	1.37	0.83	1.52	-0.88	-1.23	-1.16	-1.11	-0.33	-0.48
Naphthalene	-0.20	2.31	1.90	1.41	2.07	-0.06	-0.33	-0.27	-0.17	-0.29	-0.29
Norbornadiene	-1.04	1.62	1.24	0.65	1.38	-0.85	-1.18	-1.21	-1.07	-0.35	-0.42
Pyrazine	-0.07	2.87	2.48	1.88	2.62	0.02	-0.34	-0.25	-0.22	-0.20	-0.19
Pyridazine	-0.32	2.82	2.43	1.83	2.57	-0.02	-0.38	-0.29	-0.26	0.00	-0.26
Pyrimidine	-0.25	2.63	2.23	1.62	2.37	-0.21	-0.58	-0.53	-0.46	-0.45	-0.47
Styrene	-0.25	2.28	1.89	1.37	2.05	-0.04	-0.40	-0.34	-0.26	-0.12	-0.34
Thiophene	-1.17	1.58	1.25	0.72	1.38	-1.12	-1.41	-1.31	-1.31	-0.39	-0.45
$trans$-Dichloroethylene	-0.82	1.96	1.61	1.02	1.74	-0.87	-1.17	-1.14	-1.08	-0.47	-0.53
Trichloroethylene	-0.58	2.10	1.74	1.18	1.88	-0.60	-0.92	-0.86	-0.81	-0.36	-0.75
Uracil	-0.21	2.64	2.26	1.71	2.38	-0.04	-0.40	-0.34	-0.30	0.07	-0.30
1,2,4-Trimethylbenzene	-1.07	1.21	0.85	0.33	0.99	-1.04	-1.34	-1.29	-1.24	-0.30	-0.39
Acetone	-1.51	1.86	1.54	0.77	1.65	-1.25	-1.51	-1.56	-1.44	-0.29	-0.33
Aniline[a]	-1.13	1.29	0.92	0.39	1.06	-1.21	-1.54	-1.49	-1.43	-0.25	-0.32
Anisole	-1.09	1.40	1.02	0.48	1.17	-1.07	-1.43	-1.42	-1.31	-0.28	-0.35
cis-Butene[b,c,f]	-2.22	0.55	0.24	-0.41	0.36	-2.24	-2.49	-2.47	-2.42	-0.39	-0.45
Cyclohexene[b,c,g]	-2.07	0.75	0.29	-0.36	0.36	-1.21	-1.78	-1.85	-1.56	-0.34	-0.41
Furan	-1.76	1.07	0.76	0.18	0.86	-1.75	-1.98	-1.84	-1.93	-0.41	-0.47
m-Xylene	-1.06	1.32	0.96	0.42	1.10	-1.03	-1.35	-1.32	-1.24	-0.31	-0.39
o-Xylene	-1.12	1.22	0.86	0.32	1.01	-1.15	-1.46	-1.43	-1.35	-0.32	-0.39
Phenol	-1.01	1.50	1.12	0.59	1.26	-1.07	-1.41	-1.35	-1.29	-0.25	-0.32
Propene[a]	-1.99	0.94	0.64	0.02	0.74	-1.96	-2.15	-1.99	-2.11	-0.45	-0.51
Pyrrole [b,c,d,f]	-2.38	0.32	0.03	-0.51	0.12	-2.36	-2.51	-2.24	-2.50	-0.29	-0.35
$trans$-Butene[b,f]	-2.10	0.63	0.33	-0.29	0.44	-2.09	-2.31	-2.24	-2.25	-0.43	-0.55
Trimethylethylene[b,c,e]	-2.24	0.65	0.39	-0.13	0.49	-1.79	-1.85	-1.43	-1.87	-0.36	-0.42
CO_2[a,b,c,d]	-3.80	0.61	0.26	-0.53	0.30	-3.52	-3.78	-3.62	-3.79	-0.76	-0.76
Guanine[a]	-0.46	1.62	1.30	0.65	1.37	-0.70	-0.50	-0.96	-0.81	0.12	0.05

Table S5. (Continued.)

Molecule	Exp	LDA	BLYP	B3LYP	PBE	GSC-LDA	GSC-BLYP	GSC-B3LYP	GSC-PBE	ΔSCF-PBE	ΔSCF-B3LYP
Monofluoroethylene	-1.91	1.25	0.95	0.31	1.03	-1.97	-2.18	-2.06	-2.15	-0.47	-0.52
$trans$-1,2-difluoroethylene	-1.84	1.32	1.01	0.39	1.07	-1.95	-2.18	-2.09	-2.16	-0.50	-0.58
cis-1,2-difluoroethylene[a]	-2.18	1.14	0.82	0.19	0.89	-2.18	-2.42	-2.34	-2.40	-0.36	-0.40
1,1-Difluoroethylene[a]	-2.39	1.09	0.79	0.17	0.84	-2.15	-2.32	-2.09	-2.33	-0.42	-0.47
Trifluoroethylene[a]	-2.45	1.06	0.74	0.11	0.77	-2.30	-2.53	-2.38	-2.53	-0.41	-0.45
Tetrafluoroethylene[a,b,c]	-3.00	0.82	0.47	-0.21	0.49	-2.71	-3.04	-3.07	-3.03	-0.89	-0.91
Nitrogen	-2.20	2.18	1.88	0.98	1.92	-2.20	-2.41	-2.40	-2.41	N/A	-1.82
Formaldehyde	-0.86	2.91	2.57	1.75	2.66	-0.92	-1.18	-1.14	-1.14	N/A	-0.46
Butadiene	-0.62	2.12	1.76	1.18	1.90	-0.61	-0.93	-0.85	-0.82	N/A	-0.73
Biphenyl	-0.30	2.04	1.63	1.13	1.80	0.01	-0.40	-0.39	-0.24	N/A	-0.37
Trichloromethane	-0.35	2.47	2.22	1.55	2.31	-0.24	-0.44	-0.46	-0.38	-0.15	-0.26
Dichlorofluoromethane	-0.96	1.99	1.74	1.08	1.81	-0.90	-1.06	-1.00	-1.04	-0.36	-0.44
Dichlorodifluoromethane	-0.98	2.37	2.11	1.43	2.17	-0.61	-0.80	-0.79	-0.77	-0.42	-0.48
Dichloromethane	-1.23	1.74	1.52	0.90	1.59	-1.02	-1.08	-0.89	-1.09	-0.31	-0.38
Benzene	-1.15	1.44	1.06	0.50	1.21	-1.18	-1.52	-1.48	-1.40	-0.36	-0.42
CO	-1.80	2.24	1.94	1.12	2.00	-1.89	-2.07	-1.96	-2.08	-1.05	-1.11
Cyanogen	-0.58	3.87	3.48	2.84	3.61	-0.48	0.12	0.23	0.23	0.21	0.29
Propyne[b,c,d,e]	-2.95	0.13	0.02	-0.40	0.35	-2.29	-1.83	-1.23	-2.53	-0.40	-0.47
Butadiyne	-1.00	2.09	1.73	1.16	1.87	-0.77	-1.07	-0.96	-0.97	-0.25	-0.36
Tetramethylethylene[b,c,d,f]	-2.27	0.42	0.20	-0.31	0.28	-1.81	-1.79	-1.48	-1.86	-0.34	-0.41
Acetylene[e]	-2.60	0.57	0.32	-0.30	0.39	-2.51	-2.58	-2.36	-2.61	-0.46	-0.53
Acrylonitrile	-0.21	3.00	2.62	2.01	2.76	-0.01	-0.35	-0.27	-0.24	0.02	-0.16
1,4-Cyclohexadiene[a]	-1.75	1.05	0.67	0.02	0.81	-1.45	-1.80	-1.89	-1.68	-0.34	-0.56
Toluene	-1.11	1.39	1.01	0.46	1.16	-1.11	-1.45	-1.43	-1.33	-0.34	-0.42
Ethylbenzene	-1.17	1.37	0.99	0.47	1.14	-1.07	-1.43	-0.90	-1.31	-0.28	-0.37
Isopropylbenzene	-1.08	1.39	0.99	0.44	1.15	-1.01	-1.38	-1.41	-1.25	-0.26	-0.34
Total MAE		2.91	2.61	2.19	2.70	0.14	0.26	0.27	0.29	1.19	0.88

[a] The $-\varepsilon_{\mathrm{LUMO}+1}$ calculated with the GSC-B3LYP is taken as the EA of this molecule.

[b] The $-\varepsilon_{\mathrm{LUMO}+1}$ calculated with the GSC-BLYP is taken as the EA of this molecule.

[c] The $-\varepsilon_{\mathrm{LUMO}+1}$ calculated with the GSC-PBE is taken as the EA of this molecule.

[d] The $-\varepsilon_{\mathrm{LUMO}+1}$ calculated with the GSC-LDA is taken as the EA of this molecule.

[e] The $-\varepsilon_{\mathrm{LUMO}+2}$ calculated with the GSC-B3LYP is taken as the EA of this molecule.

[f] The $-\varepsilon_{\mathrm{LUMO}+3}$ calculated with the GSC-B3LYP is taken as the EA of this molecule.

[g] The $-\varepsilon_{\mathrm{LUMO}+4}$ calculated with the GSC-B3LYP is taken as the EA of this molecule.

Table S6: MAE between the calculated and experimental EAs for the test set of 38 molecules and the extended test set of 64 molecules with negative EAs. EAs are calculated by using the DFAs, GSC-DFAs and ΔSCF method. The data with GSC-DFAs are obtained by choosing the corrected virtual orbital as the frontier orbital to perform scaling correction. All energies are in units of eV.

	Test Set of 38 Molecules		Extended Test Set of 64 Molecules	
method	cc-pVTZ	aug-cc-pVTZ	cc-pVTZ	aug-cc-pVTZ
LDA	2.68	2.75	2.86	2.92
BLYP	2.31	2.41	2.46	2.59
B3LYP	1.66	1.82	1.81	1.99
PBE	2.40	2.53	2.57	2.70
GSC-LDA	0.21	0.13	0.23	0.14
GSC-BLYP	0.55	0.25	0.54	0.25
GSC-B3LYP	0.51	0.25	0.52	0.27
GSC-PBE	0.43	0.18	0.43	0.19
ΔSCF-PBE	0.48	0.86	0.47	1.19
ΔSCF-B3LYP	0.50	0.80	0.48	0.88

REFERENCES

Ando, N., Mitsui, M., and Nakajima, A. (2008). Photoelectron spectroscopy of cluster anions of naphthalene and related aromatic hydrocarbons. *The Journal of Chemical Physics* 128, 154318. doi:10.1063/1.2903473

Brundle, C., Robin, M., and Kuebler, N. (1972). Perfluoro effect in photoelectron spectroscopy. II. Aromatic molecules. *Journal of the American Chemical Society* 94, 1466–1475. doi:10.1021/ja00760a008

Chiu, N. S., Burrow, P. D., and Jordan, K. D. (1979). Temporary anions of the fluoroethylenes. *Chemical Physics Letters* 68, 121–126. doi:10.1016/0009-2614(79)80082-2

Chong, D. P., Gritsenko, O. V., and Baerends, E. J. (2002). Interpretation of the Kohn–Sham orbital energies as approximate vertical ionization potentials. *The Journal of Chemical Physics* 116, 1760–1772. doi:10.1063/1.1430255

Chowdhury, S., Grimsrud, E. P., Heinis, T., and Kebarle, P. (1986). Electron affinities of perfluorobenzene and perfluorophenyl compounds. *Journal of the American Chemical Society* 108, 3630–3635. doi:10.1021/ja00273a014

Chowdhury, S. and Kebarle, P. (1986). Electron affinities of di-and tetracyanoethylene and cyanobenzenes based on measurements of gas-phase electron-transfer equilibria. *Journal of the American Chemical Society* 108, 5453–5459. doi:10.1021/ja00278a014

Desfrancois, C., Periquet, V., Lyapustina, S., Lippa, T., Robinson, D., Bowen, K., et al. (1999). Electron binding to valence and multipole states of molecules: Nitrobenzene, para-and meta-dinitrobenzenes. *The Journal of Chemical Physics* 111, 4569–4576. doi:10.1063/1.479218

Dillow, G. W. and Kebarle, P. (1989). Electron affinities of aza-substituted polycyclic aromatic hydrocarbons. *Canadian Journal of Chemistry* 67, 1628–1631. doi:10.1139/v89-249

Dougherty, D., Lewis, J., Nauman, R., and McGlynn, S. (1980). Photoelectron spectroscopy of azulenes. *Journal of Electron Spectroscopy and Related Phenomena* 19, 21–33. doi:10.1016/0368-2048(80)80032-6

Dougherty, D. and McGlynn, S. (1977). Photoelectron spectroscopy of carbonyls. 1, 4-benzoquinones. *Journal of the American Chemical Society* 99, 3234–3239. doi:10.1021/ja00452a005

Frisch, M. J., Trucks, G. W., Schlegel, H. B., Scuseria, G. E., Robb, M. A., Cheeseman, J. R., et al. (2009). Gaussian 09, RevisionA.01 Wallingford, CT: Gaussian, Inc.

Fujikawa, T., Ohta, T., and Kuroda, H. (1976). X-ray photoelectron spectroscopy of the molecules containing the C≡N group. *Bulletin of the Chemical Society of Japan* 49, 1486–1492. doi:10.1246/bcsj.49.1486

Galasso, V., Colonna, F., and Distefano, G. (1977). Photoelectron spectra of 1, 2-indandione, 1, 3-indandione and heterocyclic analogues. *Journal of Electron Spectroscopy and Related Phenomena* 10, 227–237. doi:10.1016/0368-2048(77)85022-6

Heinis, T., Chowdhury, S., Scott, S. L., and Kebarle, P. (1988). Electron affinities of benzo-, naphtho-, and anthraquinones determined from gas-phase equilibria measurements. *Journal of the American Chemical Society* 110, 400–407. doi:10.1021/ja00210a015

Ikemoto, I., Samizo, K., Fujikawa, T., Ishii, K., Ohta, T., and Kuroda, H. (1974). Photoelectron spectra of tetracyanoethylene (TCNE) and tetracyanoquinodimethane (TCNQ). *Chemistry Letters* 3, 785–790. doi:10.1246/cl.1974.785

Jordan, K. D. and Burrow, P. D. (1978). Studies of the temporary anion states of unsaturated hydrocarbons by electron transmission spectroscopy. *ChemInform* 10, 341–348. doi:10.1021/ar50129a004

Khuseynov, D., Fontana, M. T., and Sanov, A. (2012). Photoelectron spectroscopy and photochemistry of tetracyanoethylene radical anion in the gas phase. *Chemical Physics Letters* 550, 15–18. doi:10.1016/j.cplett.2012.08.035

Kimura, K. (1981). *Handbook of HeI photoelectron spectra of fundamental organic molecules* (Halsted Press)

Knight, J. W., Wang, X., Gallandi, L., Dolgounitcheva, O., Ren, X., Ortiz, J. V., et al. (2016). Accurate ionization potentials and electron affinities of acceptor molecules III: A benchmark of gw methods. *Journal of Chemical Theory and Computation* 12, 615–626. doi:10.1021/acs.jctc.5b00871

Maier, J. P., Muller, J.-F., Kubota, T., and Yamakawa, M. (1975). Ionisation energies and the electronic structures of the n-oxides of azanaphthalenes and azaanthracenes. *Helvetica Chimica Acta* 58, 1641–1648. doi:10.1002/hlca.19750580619

Mei, Y., Li, C., Su, N. Q., and Yang, W. (2018). Approximating quasiparticle and excitation energies from ground state generalized Kohn–Sham calculations. *The Journal of Physical Chemistry A* 123, 666–673. doi:10.1021/acs.jpca.8b10380

Neijzen, B. and De Lange, C. (1978). Photoelectron spectroscopy of mono-and dicyanobenzenes and their perfluoro derivatives. *Journal of Electron Spectroscopy and Related Phenomena* 14, 187–199. doi:10.1016/0368-2048(78)85067-1

Ng, L., Balaji, V., and Jordan, K. D. (1983). Measurement of the vertical electron affinities of cyanogen and 2,4-hexadiyne. *Chemical Physics Letters* 101, 171–176. doi:10.1016/0009-2614(83)87365-5

Paul, G. and Kebarle, P. (1989). Electron affinities of cyclic unsaturated dicarbonyls: maleic anhydrides, maleimides, and cyclopentenedione. *Journal of the American Chemical Society* 111, 464–470. doi:10.1021/ja00184a009

Rabalais, J. (1972). Photoelectron spectroscopic investigation of the electronic structure of nitromethane and nitrobenzene. *The Journal of Chemical Physics* 57, 960–967. doi:10.1063/1.1678346

Schmidt, W. (1977). Photoelectron spectra of polynuclear aromatics. V. Correlations with ultraviolet absorption spectra in the catacondensed series. *The Journal of Chemical Physics* 66, 828–845. doi:10.1063/1.433961

Schreiber, M., Silva-Junior, M. R., Sauer, S. P., and Thiel, W. (2008). Benchmarks for electronically excited states: CASPT2, CC2, CCSD, and CC3. *The Journal of Chemical Physics* 128, 134110. doi:10.1063/1.2889385

Tozer, D. J. and De Proft, F. (2005). Computation of the hardness and the problem of negative electron affinities in density functional theory. *The Journal of Physical Chemistry A* 109, 8923–8929. doi:10.1021/jp053504y

Yang, Y., Peng, D., Lu, J., and Yang, W. (2014). Excitation energies from particle-particle random phase approximation: Davidson algorithm and benchmark studies. *Journal of Chemical Physics* 141, 124104–124104. doi:10.1063/1.4895792

Zhang, D., Yang, X., Zheng, X., and Yang, W. (2018). Accurate density functional prediction of molecular electron affinity with the scaling corrected Kohn–Sham frontier orbital energies. *Molecular Physics* 116, 927–934. doi:10.1080/00268976.2017.1382738

Zlatkis, A., Lee, C., Wentworth, W., and Chen, E. (1983). Constant current linearization for determination of electron capture mechanisms. *Analytical Chemistry* 55, 1596–1599. doi:10.1021/ac00260a034
